# The MAPK kinase BcMkk1 suppresses oxalic acid biosynthesis via impeding phosphorylation of BcRim15 by BcSch9 in *Botrytis cinerea*

**DOI:** 10.1371/journal.ppat.1007285

**Published:** 2018-09-13

**Authors:** Yanni Yin, Sisi Wu, Chaonan Chui, Tianling Ma, Huixian Jiang, Matthias Hahn, Zhonghua Ma

**Affiliations:** 1 State Key Laboratory of Rice Biology, Institute of Biotechnology, Zhejiang University, Hangzhou, China; 2 Department of Biology, Kaiserslautern University, Kaiserslautern, Germany; Nanjing Agricultural University, CHINA

## Abstract

The mitogen-activated protein kinase (MAPK) cassette of the cell wall integrity (CWI) pathway is primarily responsible for orchestrating changes of cell wall. However, functions of this cassette in other cellular processes are not well understood. Here, we found that the *Botrytis cinerea* mutant of MAPK kinase (BcMkk1) displays more serious defects in mycelial growth, conidiation, responses to cell wall and oxidative stresses, but possesses less reduced virulence than the mutants of its upstream (BcBck1) and downstream (BcBmp3) kinases. Interestingly, BcMkk1, but not BcBck1 and BcBmp3, negatively regulates production of oxalic acid (OA) and activity of extracellular hydrolases (EHs) that are proposed to be virulence factors of *B*. *cinerea*. Moreover, we obtained evidence that BcMkk1 negatively controls OA production via impeding phosphorylation of the Per-Arnt-Sim (PAS) kinase BcRim15 by the Ser/Thr kinase BcSch9. In addition, the fungal Pro40 homolog BcPro40 was found to interact simultaneously with three MAPKs, implying that BcPro40 is a scaffold protein of the CWI pathway in *B*. *cinerea*. Taken together, results of this study reveal that BcMkk1 negatively modulates virulence via suppressing OA biosynthesis in *B*. *cinerea*, which provides novel insight into conserved and species-specific functions of the MAPK kinase in fungi.

## Author summary

*Botrytis cinerea* causes pre- and postharvest diseases in more than 200 economically important crops. In this study, the roles of cell wall integrity (CWI)-related MAPK kinase BcMkk1in regulating *B*. *cinerea* virulence were investigated using genetic and biochemical approaches. We found that the MAPK kinase BcMkk1 positively regulates virulence via the CWI pathway. Unexpectedly, BcMkk1 also negatively regulates fungal virulence via restraining oxalic acid production, by impeding phosphorylation of the PAS kinase BcRim15 mediated by the kinase BcSch9. To our knowledge, this is the first report that a MAPK kinase can negatively modulate fungal virulence on host plants. Our results provide novel insight into biological functions of a MAPK kinase in fungal pathogenesis.

## Introduction

The cell wall integrity (CWI) signaling pathway is required for remodeling of the fungal cell wall in a highly regulated and polarized manner during growth, morphogenesis, and responses to environmental challenges [1]. This pathway has been well understood in *Saccharomyces cerevisiae* [2–4]. In the yeast, cell surface sensors Wsc1, -2, -3, Mid2 and Mtl1 respond to cell wall stress stimuli by activating the small G protein Rho1 via its guanosine nucleotide exchange factor Rom2. Rho1 then (interacts with and) activates Pkc1, which in turn triggers a mitogen-activated protein kinase (MAPK) cascade consisting of the MEKK Bck1, a pair of redundant MEKs (Mkk1 and Mkk2) and Mpk1, also named Slt2. Activation of the MAPK cascade transmits signals to several transcription factors including Swi4, Swi6 and Rlm1, which activate expression of genes involved either in cell wall biosynthesis and remodeling or in cell cycle progression [2–4].

The core components of the CWI pathway are well conserved in fungi and its biological functions in regulating the integrity of cell wall have been reported in several filamentous fungi, including *Magnaporthe oryzae*, *Claviceps purpurea*, *Aspergillus nidulans* and *Alternaria alternata* [5–8]. However, our knowledge about functions of the CWI pathway in other cellular processes is limited. In the plant pathogen *Fusarium graminearum* and human pathogens *Candida albicans*, *Cryptococcus neoformans* and *Aspergillus fumigatus*, the MAPKs of CWI pathway are required not only for the cell wall integrity, but also for virulence and osmotic stress response [9–12]. In *Colletotrichum lagenarium*, this pathway seems not involved in cell wall integrity, but plays an important role in appressorium differentiation and infection [13]. In *Sordaria macrospora*, the CWI pathway regulates sexual development and hyphal fusion as well as cell wall integrity [14]. These studies indicated that the CWI pathway may possess diverse functions in pathogenic fungi, which are not known in *S*. *cerevisiae*.

Oxalic acid (OA) as a by-product of metabolism is produced and secreted by many filamentous fungi [15–17]. In plant pathogenic fungi, OA has been found to play multiple functions in pathogenesis mainly including acidifying host tissues and enhancing the activity of extracellular hydrolases (EHs) [18–20], chelating Ca^2+^ in plant cell wall and weakening plant cell wall [21,22], and reducing the oxidative burst [23,24]. In *Botrytis cinerea*, *Sclerotinia sclerotiorum*, *Aspergillus niger* and other filamentous ascomycetous fungi, OA is mainly produced via oxaloacetate acetylhydrolase (OAH) which catalyses the hydrolytic cleavage of oxaloacetate to acetate and oxalate [15,22,25–28]. Characterization of Oah mutants in *S*. *sclerotiorum* [29,30], *B*. *cinerea* [31,32] and *Cryphonectria parasitica* [22] has found that the mutants caused disease and do produced limited lesions on some host plants, and suggested that OA has a role of tissue acidification in lesion extension.

*B*. *cinerea*, a necrotrophic plant pathogen, causes gray mold diseases in numerous dicotyledonous plant species including high-value crops such as grapevine, strawberry, raspberry, and ornamental flowers [33]. Moreover, *B*. *cinerea* attacks the hosts during both pre- and post-harvest resulting in considerable economic loss, and global expenses of *Botrytis* control easily surmount €1 billion per annum [34]. A previous study found that the BcBmp3 (the ortholog of Slt2) mutant did not show increased sensitivity to cell wall-damaging agents glucanase, calcofluor white (CFW) and nikkomycin Z, indicating that the role of CWI in *B*. *cinerea* might be different from what is known in the budding yeast and other fungi [35]. Thus, we initiated this project for exploring the functions of the CWI pathway in *B*. *cinerea*. Unexpectedly, we found that the *BcMKK1* deletion mutant (ΔBcMkk1) showed slower mycelial growth, higher sensitivity to cell wall and oxidative stresses, but was less impaired in virulence than ΔBcBck1 (the upstream kinase of BcMkk1) and ΔBcBmp3 (the downstream kinase of BcMkk1). Deletion of *BcMKK1* caused a dramatic increase in the activity of several EHs and OA production. Further deletion of OA synthase-encoding gene *BcOAH* in ΔBcMkk1 led to significantly reduced EH activity and OA production revealing that BcOah is genetically associated with OA production and EH activity. Moreover, a PAS (Per-Arnt-Sim) kinase BcRim15 was found to be a novel downstream component of BcMkk1 which is involved in OA biosynthesis and pathogenesis. BcMkk1 negatively regulated OA biosynthesis via impeding phosphorylation of the PAS kinase BcRim15 mediated by kinase BcSch9. The novel finding indicates that BcMkk1 has negative roles in regulating virulence of *B*. *cinerea*, which extends our understanding of the CWI-related MAPK pathway in pathogenic fungi.

## Results

### BcMkk1 is more important than its up- and down-stream kinases for hyphal growth and asexual development

Based on the amino acid sequences of MAPKs Bck1, Mkk1/2 and Slt2 from the budding yeast *S*. *cerevisiae*, BcBck1 (Bcin02g06590.1), BcMkk1 (Bcin03g07190.1) and BcBmp3 (Bc09g02430.1) were retrieved from the genome of *B*. *cinerea* (http://fungidb.org/fungidb/). BcBck1, BcMkk1 and BcBmp3 are highly homologous to their counterparts from other fungal species (S1 Fig). To investigate the functions of BcMkk1, BcBck1and BcBmp3 in *B*. *cinerea*, we generated gene deletion mutants using a homology recombination strategy (S2A Fig). The resulting hygromycin-resistant transformants of each gene were screened by PCR with the primer pairs listed in S1 Table (S2B Fig). In addition, Southern blot assays verified that each mutant is a null mutant resulting from homologous recombination events at the corresponding gene locus (S2C Fig). Furthermore, to confirm that phenotypic changes observed in ΔBcMkk1 were due to the deletion of *BcMKK1*, the mutant was complemented with a full-length wild-type *BcMKK1* gene. The complemented strain ΔBcMkk1-C contained a single copy of *BcMKK1* inserted into the genome of ΔBcMkk1 (S2B Fig). Similarly, the mutants ΔBcBck1 and ΔBcBmp3 were also complemented, and the complemented strains were confirmed by PCR assays (S2B Fig).

**Fig 1:**
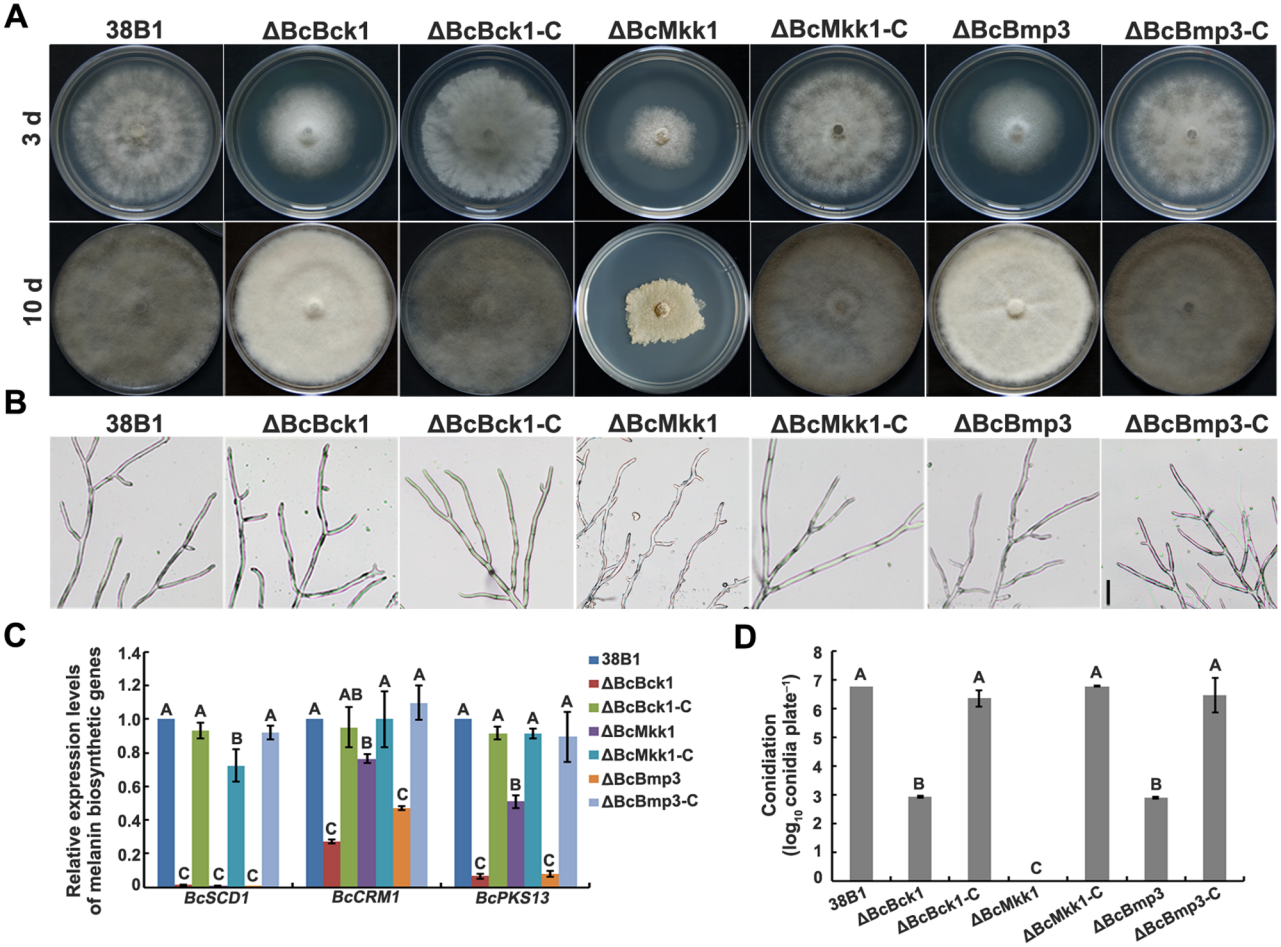
Deletion of *BcBCK1*, *BcMKK1* or *BcBMP3* led to reduced mycelial growth, conidiation and melanin production in *B*. *cinerea*. (A) Colony morphology of 38B1, ΔBcBck1, ΔBcMkk1, ΔBcBmp3, ΔBcBck1-C, ΔBcMkk1-C and ΔBcBmp3-C on PDA after 3 and 10 days of incubation at 25°C. (B) Hyphae of ΔBcMkk1, but not ΔBcBck1 and ΔBcBmp3, were wavy. Bar = 100 μm. (C) Comparisons of the transcript levels of three melanin biosynthesis related genes *CMR1*, *SCD1* and *PKS13* among the above strains. The expression level of each gene in 38B1 was referred to 1. Values on the bars followed by the same letter are not significantly different at *P* = 0.05. (D) Comparisons in conidiation among the above strains on PDA plates after 10 days of incubation. Values on the bars followed by the same letter are not significantly different at *P* = 0.05.

The deletion mutants ΔBcBck1, ΔBcMkk1 and ΔBcBmp3 grew significantly slower and exhibited less aerial hyphae than the wild-type progenitor 38B1 on potato dextrose agar (PDA) plate (Fig 1A). Interestingly, the mutant ΔBcMkk1 exhibited a slower growth rate than ΔBcBck1 and ΔBcBmp3 after 3 days of incubation on PDA. Further microscopic examination revealed that hyphae of ΔBcMkk1 were twisted, but hyphae of 38B1, ΔBcBck1 and ΔBcBmp3 grew relatively smooth and straight (Fig 1B). These results indicate that BcMkk1 plays more important roles in vegetative growth than its upstream and downstream kinases BcBck1 and BcBmp3, respectively.

Colony morphology examination showed that ΔBcBck1, ΔBcMkk1 and ΔBcBmp3 produced dramatically less melanin in comparison with the wild type (Fig 1A), as previously shown for ΔBcBmp3 by Liu et al. [36]. This observation was further confirmed by determining the expression levels of three melanin biosynthesis-related genes *BcSCD1*, *BcCMR1* and *BcPKS13* [36,37] in these mutants. Quantitative reverse transcription PCR (qRT-PCR) assays showed that all three genes were down-regulated significantly in the three mutants (Fig 1C), demonstrating that MAPKs BcBck1, BcMkk1 and BcBmp3 regulate melanin biosynthesis in *B*. *cinerea*.

Deletion of *BcBCK1*, *BcMKK1* or *BcBMP3* led to seriously impaired conidiation. As shown in Fig 1A and 1D, ΔBcMkk1 totally lost the ability in conidiation, while ΔBcBck1 and ΔBcBmp3 produced less conidia in comparison with the wild type after incubation on PDA for 10 days. The defects of mycelial growth, melanin production and conidiation in ΔBcBck1, ΔBcMkk1and ΔBcBmp3 were restored by genetic complementation of the mutants with the wild-type *BcBCK1*, *BcMKK1* and *BcBMP3*, respectively (Fig 1A, 1C and 1D).

### Involvement of BcBck1, BcMkk1 and BcBmp3 in the responses to cell wall and oxidative stresses

**Fig 2:**
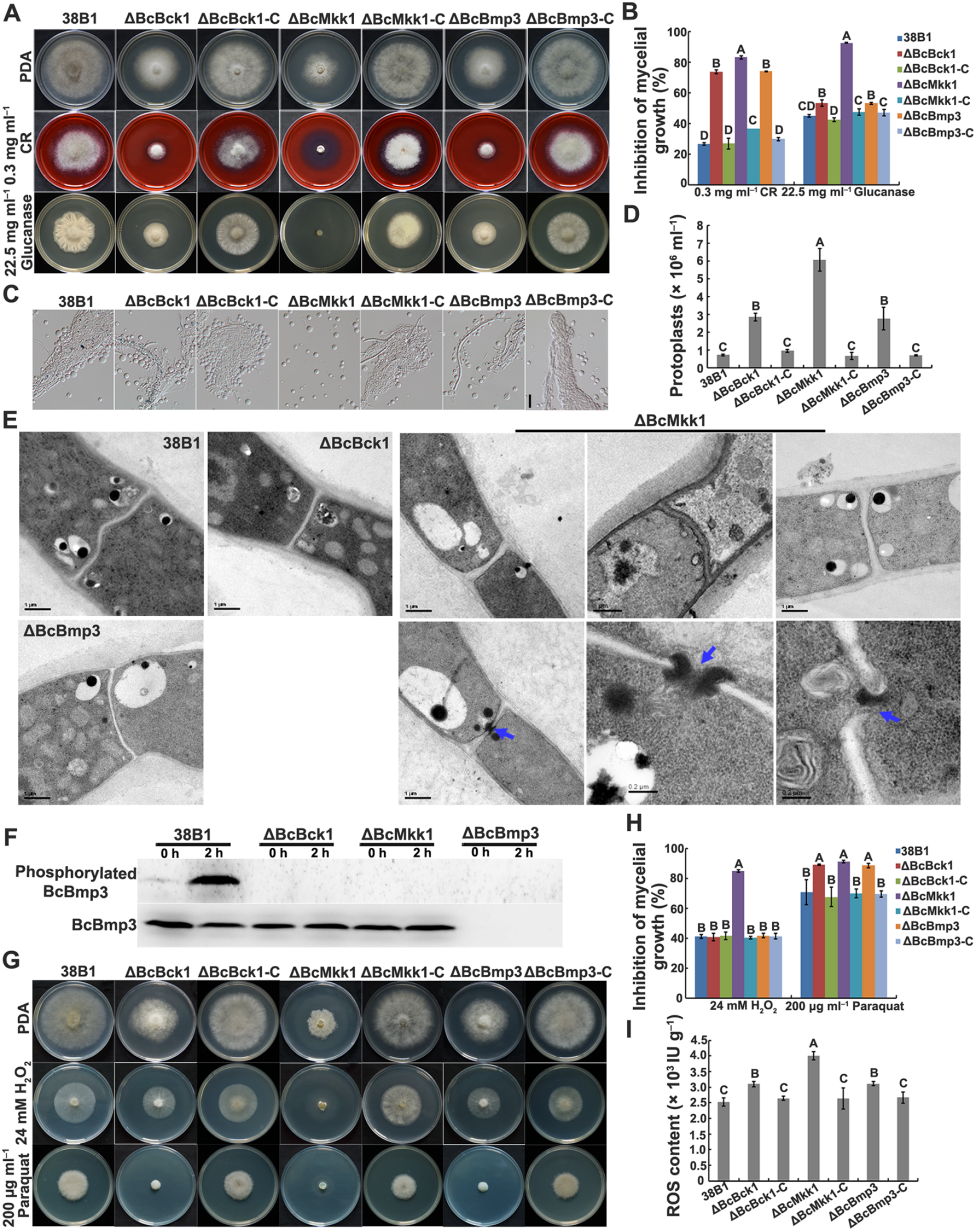
ΔBcMkk1 exhibited more serious defects in response to cell wall and oxidative stresses than ΔBcBck1 and ΔBcBmp3. (A) Sensitivity of 38B1, ΔBcBck1, ΔBcMkk1, ΔBcBmp3, ΔBcBck1-C, ΔBcMkk1-C and ΔBcBmp3-C to Congo Red (CR) and glucanase after incubation at 25°C for 3 days. (B) Mycelial growth inhibition of each strain shown in (A). Values on the bars followed by the same letter are not significantly different at *P* = 0.05. (C) Mycelia of ΔBcMkk1 not ΔBcBck1 and ΔBcBmp3 were well digested and released abundant protoplasts after treatment with glucanex at 25°C for 2 h. Bar = 20 μm. (D) Protoplast amount of each strain shown in (C). Values on the bars followed by the same letter are not significantly different at *P* = 0.05. (E) ΔBcMkk1, but not ΔBcBck1 and ΔBcBmp3, exhibited thickened septa (up panels of ΔBcMkk1) and occasionally with a larger central pore indicated by blue arrows (low panels of ΔBcMkk1) through transmission electron microscopic examination. (F) Comparisons of BcBmp3 phosphorylation among the above strains. After grown in PDB for 2 days, mycelia of each strain treated with 0.3 mg ml^–1^ of CR for 0 and 2 h were collected for protein extraction. BcBmp3 and phosphorylated BcBmp3 proteins were detected with the anti-Mpk1 and phospho-p44/42 MAPK antibodies, respectively. (G) Sensitivity of the above strains to H_2_O_2_ and paraquat after incubation at 25°C for 3 days. (H) Mycelial growth inhibition of each strain shown in (G). Values on the bars followed by the same letter are not significantly different at *P* = 0.05. (I) ROS content in vegetative hyphae of each strain. Values on the bars followed by the same letter are not significantly different at *P* = 0.05.

To investigate functions of the BcBck1-BcMkk1-BcBmp3 cassette in cell wall integrity, we examined sensitivity of ΔBcBck1, ΔBcMkk1 and ΔBcBmp3 to cell wall-damaging agents and -degrading enzyme. As shown in Fig 2A–2D, these mutants showed increased sensitivity to Congo Red (CR) and glucanase, and generated more protoplasts than the wild type after treatment with glucanex at 25°C for 2 h. Notably, ΔBcMkk1 displayed more susceptibility to cell wall stress in comparison with ΔBcBck1 and ΔBcBmp3. Furthermore, deletion of *BcMKK1* caused thickened septa (Fig 2E, up panels of ΔBcMkk1), occasionally with larger central pores (Fig 2E, indicated with blue arrows in low panels of ΔBcMkk1), whereas deletion of *BcBCK1* or *BcBMP3* did not result in recognizable changes in septum morphology when examined with transmission electron microscopy (Fig 2E upper panel).

In the budding yeast, Bck1 phosphorylates Mkk1/Mkk2, then Mkk1/2 phosphorylates the MAP kinase Slt2, and finally the activated Slt2 interacts with target proteins such as the transcription factor Rlm1 [2]. To explore relationships between BcBck1, BcMkk1 and BcBmp3, phosphorylation profiling of BcBmp3 was performed. In the wild-type 38B1, the phosphorylation level of BcBmp3 was up-regulated significantly by the CR treatment (Fig 2F). In contrast, phosphorylation of BcBmp3 was not detected in ΔBcBck1 and ΔBcMkk1 either treated or untreated with CR (Fig 2F), indicating that phosphorylation of BcBmp3 is dependent on BcBck1 and BcMkk1, and these two MAPKs function upstream of BcBmp3 in *B*. *cinerea*.

Previous studies have shown that the fungal CWI pathway might be involved in oxidative stress. Thus, we also determined sensitivity of ΔBcBck1, ΔBcMkk1 and ΔBcBmp3 to hydrogen peroxide and paraquat since these compounds can elicit different oxidative stresses. As shown in Fig 2G and 2H, ΔBcMkk1 displayed increased sensitivity to both hydrogen peroxide and paraquat, but ΔBcBck1 and ΔBcBmp3 showed elevated sensitivity only to paraquat, but not to hydrogen peroxide, in comparisons with the wild type. Further ROS content assay showed that deletion of *BcBCK1*, *BcMKK1* and *BcBMP3* all caused ROS accumulation in *B*. *cinerea* cells, moreover the level of ROS in ΔBcMkk1 is higher than those in ΔBcBck1 and ΔBcBmp3 (Fig 2I). These results indicated that although the BcBck1-BcMkk1-BcBmp3 cassette regulates the response to oxidative stress, BcMkk1 possesses an addition role in this process.

### ΔBcMkk1 exhibited higher virulence than ΔBcBck1 and ΔBcBmp3

**Fig 3:**
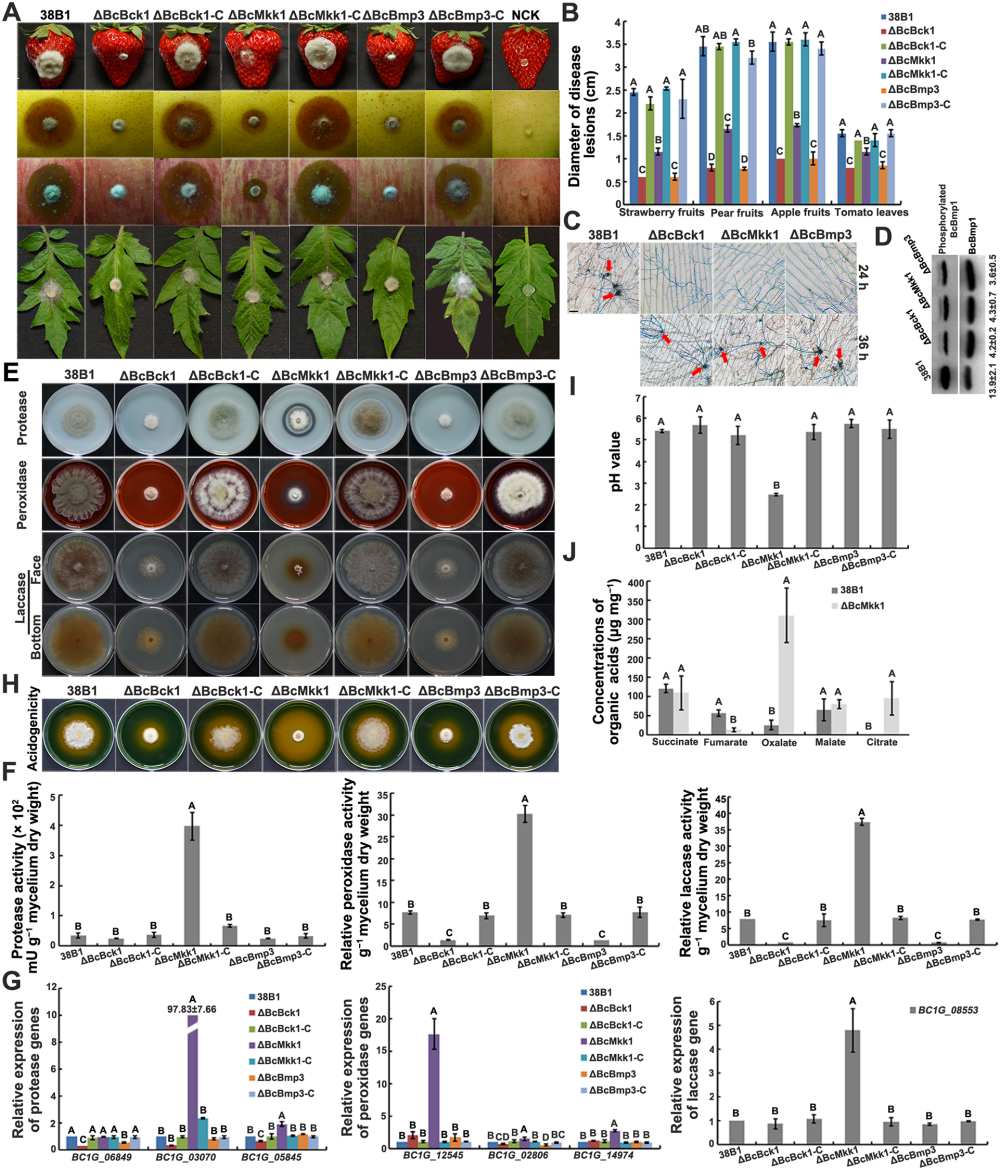
ΔBcMkk1 displayed higher virulence, increased activity of extracellular hydrolases (EHs) and more oxalic acid than ΔBcBck1 and ΔBcBmp3. (A) Pathogenicity of each strain on strawberry, pear and apple fruits, and tomato leaves. Agar plugs without fungal mycelia were used as negative controls (NCKs). Disease symptoms were photographed 60 h post inoculation (hpi). (B) Diameters of disease lesions of each strain shown in (A). Values on the bars followed by the same letter are not significantly different at *P* = 0.05. (C) Onion penetration assays of 38B1, ΔBcBck1, ΔBcMkk1 and ΔBcBmp3. Infection structures (indicated by red arrows) were stained with aniline blue 24 and 36 hpi. Bar = 20 μm. (D) Comparisons of the phosphorylation level of MAPK BcBmp1 in the Fus3/Kss1 pathway among the above strains. BcBmp1 and phosphorylated BcBmp1 proteins were detected using the p44/42 MAPK and phospho-p44/42 MAPK antibodies, respectively. The intensities of the western blotting bands were quantified with the programme IMAGE QUANT TL. The intensity of phosphorylated BcBmp1 band for each strain is relative to that of BcBmp1 band. (E) ΔBcMkk1, but not ΔBcBck1 and ΔBcBmp3, exhibited obvious degradation halo on each enzyme activity-detecting medium. Photographs were taken 3 days after inoculation (dai) for laccase detection and 5 dai for protease and peroxidase detection. (F) Activity of EHs was measured after grown in CM for 5 days. Values on the bars followed by the same letter are not significantly different at *P* = 0.05. (G) The transcription of protease, peroxidase and laccase genes in each strain. The expression level of each gene in 38B1 was referred to 1. Values on the bars followed by the same letter are not significantly different at *P* = 0.05. (H) ΔBcMkk1 produced more acidic compounds than ΔBcBck1 and ΔBcBmp3 on PDA amended with 0.05% bromothymol blue indicating acid production. (I) pH value of the 5-day-old culture supernatant of each strain grown in PDB. Values on the bars followed by the same letter are not significantly different at *P* = 0.05. (J) Comparisons of organic acid concentrations between 38B1 and ΔBcMkk1 after incubation in PDB for 5 days. Values on the bars followed by the same letter are not significantly different at *P* = 0.05.

To analyze the function of these three *B*. *cinerea* MAPKs in pathogenicity, infection assays with different plant tissues were conducted. As shown in Fig 3A and 3B, deletion of *BcBCK1*, *BcMKK1* or *BcBMP3* led to dramatically decreased virulence on strawberry, pear and apple fruits and tomato leaves 60 h after inoculation. Surprisingly, ΔBcMkk1 exhibited higher virulence than ΔBcBck1 and ΔBcBmp3 (Fig 3A and 3B), although ΔBcMkk1 displayed more serious defects than the latter in mycelial growth, conidiation, sensitivity to cell wall and oxidative stresses (Figs 1 and 2).

Appressoria produced by germ tubes and infection cushions formed by hyphae play a key role in *B*. *cinerea* penetration into the host [38,39]. To analyze the virulence defects of the mutants in details, onion penetration assays were performed. As shown in Fig 3C, hyphae of ΔBcBck1, ΔBcMkk1 and ΔBcBmp3 revealed a delay in penetration of killed onion epidermis. After 24 h of incubation, the wild-type hyphae formed highly melanized “specialized hyphal networks” or “clumps of hyphae” called infection cushions, whereas similar infection structures could not be observed from three mutants until 36 h of incubation. More importantly, ΔBcMkk1 formed similar infection structures as ΔBcBck1 and ΔBcBmp3 after 36 h of incubation. Previous studies found that MAPK Fus3/Kss1 plays important roles in infection structure formation in phytopathogenic fungi [40–43]. Thus, we also determined the phosphorylation BcBmp1 (the ortholog of Fus3) in ΔBcBck1, ΔBcBMkk1 and ΔBcBmp3. Western blotting assay showed that the phosphorylation of BcBmp1 was reduced to a similar level in ΔBcBck1, ΔBcMkk1 and ΔBcBmp3 (Fig 3D).

### BcMkk1 negatively regulates oxalic acid biosynthesis in *B*. *cinerea*

To further explore different regulatory mechanisms of BcBck1, BcMkk1 and BcBmp3 in pathogenesis, we determined the activity of secreted proteases, peroxidases and laccases for these mutants because previous studies have reported that extracellular hydrolases secreted by phytopathogenic *B*. *cinerea* favor fungal penetration and colonization in the susceptible plant [44,45]. As shown in Fig 3E, ΔBcMkk1 generated obvious degradation haloes in skimmed milk agar medium for protease assay, CM supplemented with CR for peroxidase detection and Bavendamm′s medium for determination of laccase. In contrast, degradation haloes were not observed from the wild type, ΔBcBck1 and ΔBcBmp3 under the same conditions (Fig 3E). Further hydrolase activity assays confirmed that deletion of *BcMKK1* caused increased activity of these three hydrolases, whereas deletion of *BcBCK1* or *BcBMP3* led to reduced hydrolase activity (Fig 3F). Consistent with these results, the expression levels of genes encoding three aspartic proteases (Bcin02g08590: Bcap2, Bcin12g02040: Bcap8, the most highly expressed secreted protein [46], and Bcin01g01190: Bcap3), two cytP450 monooxygenases (Bcin03g00440, Bcin07g02170.1), a secreted peroxidase (Bcin03g07850.1) and a secreted laccase (Bcin14g02510.1: Bclcc2, required for degradation of resveratrol [47]) were significantly up-regulated in ΔBcMkk1, but partially down-regulated in ΔBcBck1 and ΔBcBmp3 (Fig 3G).

Previous studies have reported that ambient pH could regulate expression of secreted proteins in *B*. *cinerea*, and proteases, peroxidases and laccases have high activity at pH between 3.0 and 4.5 [19,48,49]. Therefore, we were interested in evaluating the ability of acid production for each mutant. As shown in Fig 3H, ΔBcMkk1 dramatically changed the color of the pH-indicating medium (PDA containing 0.05% bromothymol blue as pH indicator) from green to yellow denoting the production of acid(s), in contrast, ΔBcBck1, ΔBcBmp3 and the wild type exhibited less yellowing medium indicating they produced less amount of acid(s). In addition, we determined the pH value for each mutant after incubation in potato dextrose broth (PDB) for 5 days. The pH value of the culture supernatant of ΔBcMkk1 was significantly lower than that of the wild type, ΔBcBck1, ΔBcBmp3, and the complemented strain ΔBcMkk1-C (Fig 3I), indicating strongly that deletion of *BcMKK1* led to increased secretion of acid compound(s). Organic acids including citric, malic, succinic, oxalic and fumaric acids are secreted by *B*. *cinerea in vitro* and *on planta* [50,51], we further measured organic acid secretion for ΔBcMkk1 after incubation in PDB for 5 days. Compared to the wild type, ΔBcMkk1 secreted much more oxalate and less fumarate (Fig 3J). In addition, ΔBcMkk1 secreted a tiny amount of citrate, whereas citrate was under a detectable level in the wild type (Fig 3J). Similar amounts of succinate and malate were measured in both strains (Fig 3J). These results indicate that oxalate plays a crucial role for ΔBcMkk1 in acidification of medium.

**Fig 4:**
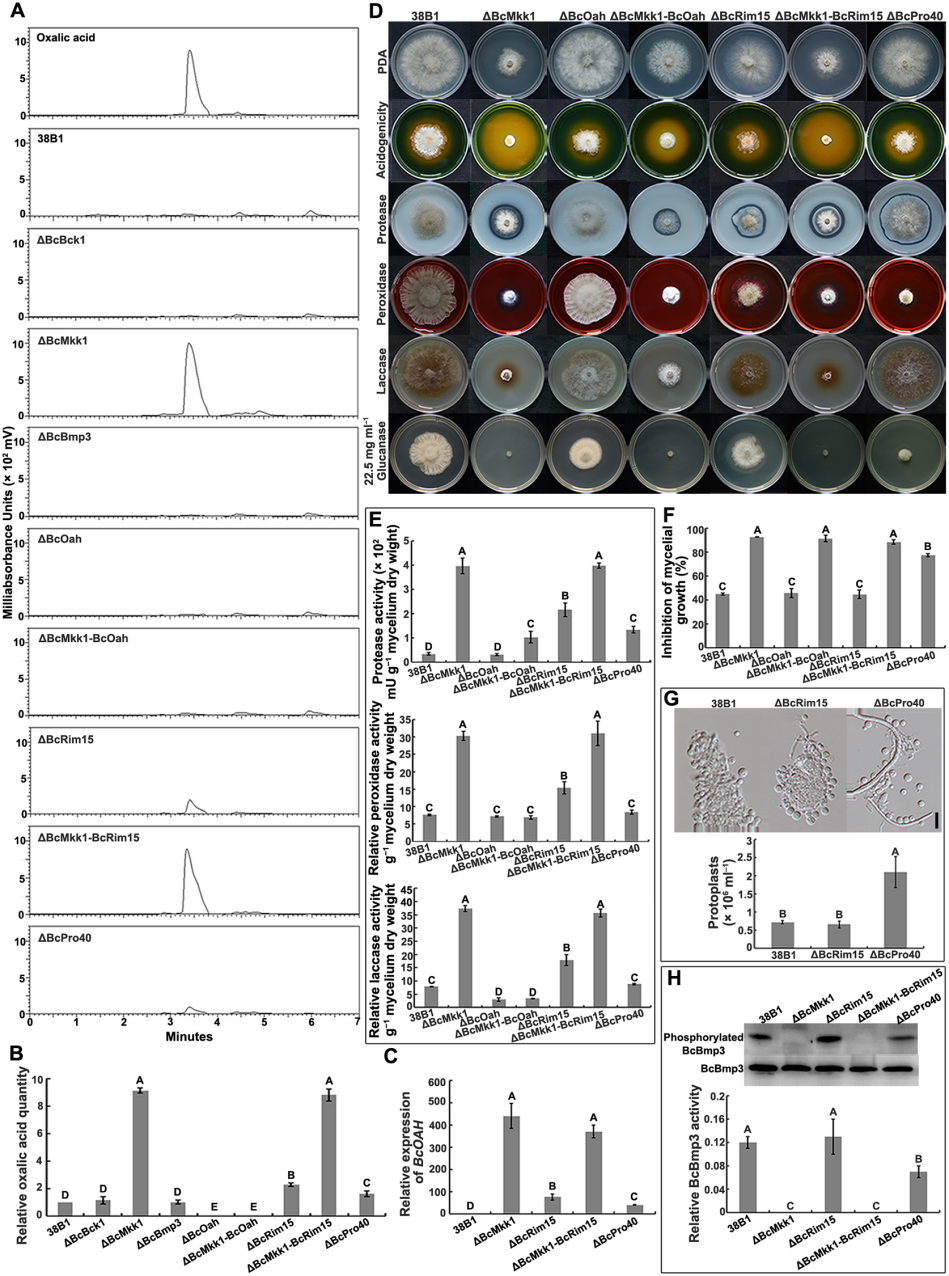
BcMkk1 negatively regulated oxalic acid (OA) biosynthesis via BcRim15. (A) HPLC profiles of the standard OA and the compound(s) in the culture supernatant of each strain. (B) Quantification of OA production for each strain in (A). The peak area in 38B1 was referred to 1. Values on the bars followed by the same letter are not significantly different at *P* = 0.05. (C) Comparisons in the expression level of *BcOAH* among 38B1, ΔBcRim15, ΔBcMkk1-BcRim15 and ΔBcPro40. The expression level in 38B1 was referred to 1. Values on the bars followed by the same letter are not significantly different at *P* = 0.05. (D) Deletion of *BcOAH* in the ΔBcMkk1 background resulted in dramatically reduced OA production and activity of EHs. (E) Activity of EHs was measured after grown in CM for 5 days. Values on the bars followed by the same letter are not significantly different at *P* = 0.05. (F) Mycelial growth inhibition of each strain grown on PDA amended with glucanase in (D). Values on the bars followed by the same letter are not significantly different at *P* = 0.05. (G) BcBmp3 phosphorylation in each strain. After grown in PDB for 2 days, mycelia of each strain treated with 0.3 mg ml^–1^ of CR for 2 h were collected for protein extraction. BcBmp3 and phosphorylated BcBmp3 proteins were detected with the anti-Mpk1 and phospho-p44/42 MAPK antibodies, respectively. The intensities of the western blotting bands were quantified with the programme IMAGE QUANT TL. The intensity of phosphorylated BcBmp3 band for each strain is relative to that of BcBmp3 band. Values on the bars followed by the same letter are not significantly different at *P* = 0.05. (H) Protoplast amount of each strain after treatment with glucanex at 25°C for 2 h. Bar = 20 μm. Values on the bars followed by the same letter are not significantly different at *P* = 0.05.

To further verify and explore the acidogenesis mechanism of ΔBcMkk1, we determined OA production in PDB of each mutant using high-performance liquid chromatography (HPLC). As shown in Fig 4A and 4B, ΔBcMkk1 produced OA dramatically higher than the wild type, ΔBcBck1 and ΔBcBmp3 did. Oxaloacetate acetylhydrolase (OAH) responsible for hydrolysis of oxaloacetate is regarded as the especially important enzyme for OA biosynthesis in *B*. *cinerea* [15]. Therefore, we generated a *BcOAH* deletion mutant (ΔBcOah) and *BcMKK1*-*BcOAH* double mutant (ΔBcMkk1-BcOah). As expected, both ΔBcMkk1-BcOah and ΔBcOah were unable to produce detectable OA (Fig 4A and 4B). Additionally, qRT-PCR assays showed that the transcript level of *BcOAH* in ΔBcMkk1 was 440-fold higher than that in the wild type (Fig 4C). Consistently, ΔBcMkk1-BcOah exhibited significantly decreased acid production indicated by the PDA medium supplement with a pH-indicator, and declined activity of protease, peroxidase and laccase (Fig 4D and 4E). These results strongly indicate that BcMkk1 negatively modulated OA production in *B*. *cinerea*.

### BcMkk1 negatively regulates oxalic acid biosynthesis via impeding phosphorylation of BcRim15 mediated by BcSch9

Given that BcMkk1 has an additional function in regulating OA production in comparisons with its upstream and downstream kinases BcBck1 and BcBmp3, we tried to identify BcMkk1-interacting proteins via affinity capture assay. Briefly, BcMkk1 fused with GFP was transformed into ΔBcMkk1 resulting in the complemented strain ΔBcMkk1-C (S3 Fig). The proteins extracted from ΔBcMkk1-C and ‘captured’ with the GFP antibody were further analyzed by mass spectrometry. Proteins extracted from a strain transformed with GFP alone were used as negative controls. In this affinity capture assay, in addition to the MAPKs of the CWI, four other kinases (BcHog1, BcRim15, BcSte20 and BcSch9) were identified as potential BcMkk1-interacting proteins (S2 Table). To test whether the four kinases are involved in regulating OA production, we obtained the deletion mutant for each of these kinase genes. Determination of OA production and protease activity showed that only a mutant deficient in BcRim15 (Bcin15g00280) displayed a significant increase in OA production and protease activity (Fig 4D and 4E; S4 Fig). In *S*. *cerevisiae*, the PAS kinase Rim15 is proposed to integrate signals from different nutrient-sensing pathways and to control transcriptional reprogramming upon nutrient depletion [52,53]. HPLC detection of OA quantity and transcript level of *BcOAH* in ΔBcRim15 further confirmed that BcRim15 was involved in OA production (Fig 4A–4C). The double deletion mutant ΔBcMkk1-BcRim15 exhibited OA accumulation and elevated activity of extracellular hydrolases as well as ΔBcMkk1 (Fig 4A–4E), indicating BcMkk1 and BcRim15 may function in the same pathway in regulation of OA production.

**Fig 5:**
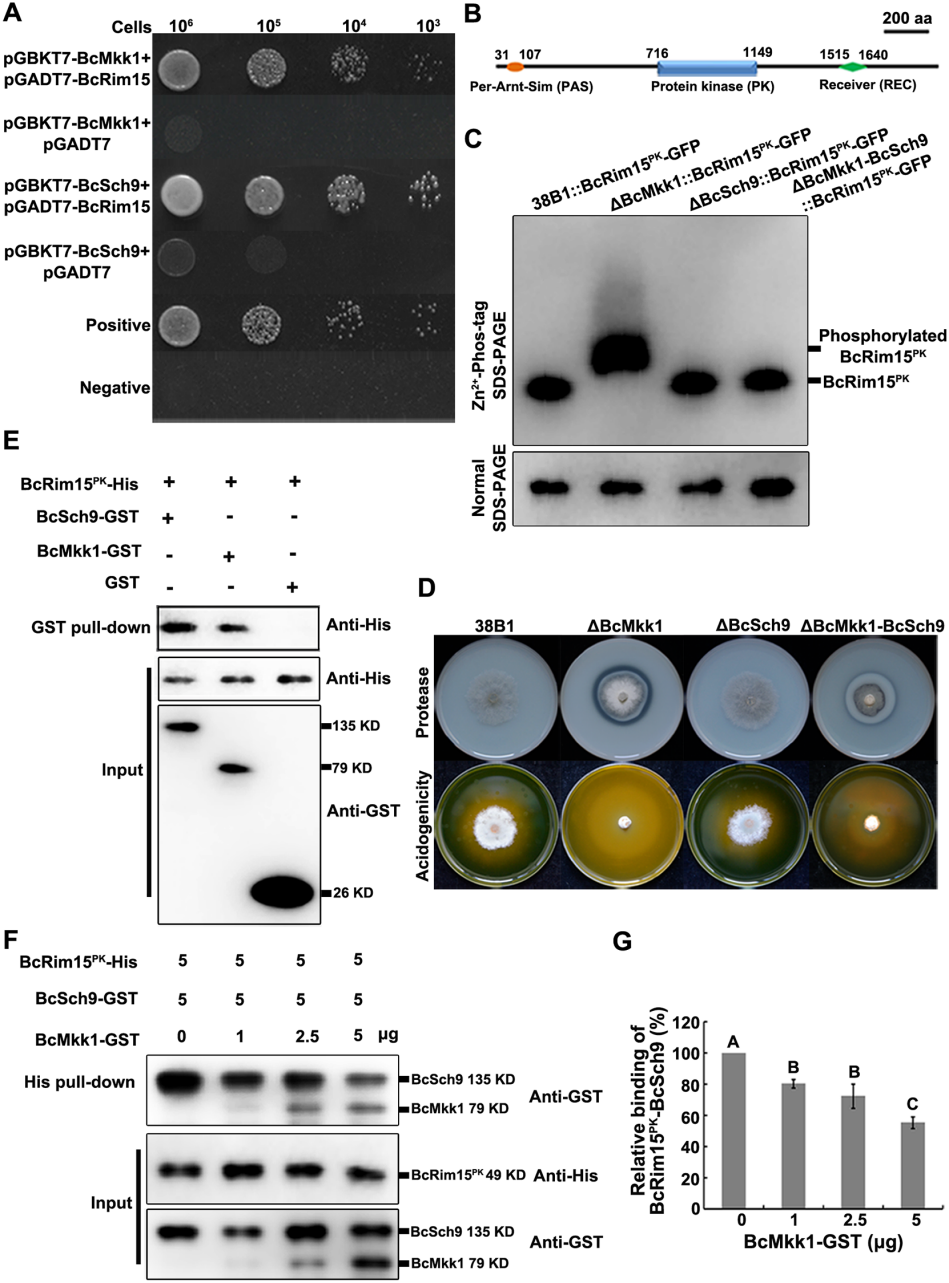
BcMkk1 impedes BcRim15 phosphorylation mediated by BcSch9. (A) The interactions of BcRim15 with BcMkk1 and BcSch9 were verified by the Y2H assays. Serial concentrations of yeast cells were drop-plated on SD-Leu-Trp-His plates. A pair of plasmids pGBKT7-53 and pGADT7 was used as a positive control. A pair of plasmids pGBKT7-Lam and pGADT7 was used as a negative control. (B) Domain architecture of the BcRim15 identified by Pfam (http://pfam.xfam.org/). (C) Phosphorylation of BcRim15^PK^ (the protein kinase domain of BcRim15) in ΔBcMkk1 was dramatically higher than that in the wild type, and was reduced in the double mutant ΔBcMkk1-BcSch9. (D) Acid production and protease activity of each strain. (E) Both BcMkk1 and BcSch9 bind directly to BcRim15^PK^. Proteins associated with the GST beads or in the total proteins before GST pull-down (input) were probed by western blot analysis using the monoclonal mouse anti-His or anti-GST antibodies. (F) BcMkk1 inhibits the interaction of BcSch9 with BcRim15^PK^. BcRim15^PK^ bound to Ni sepharose beads was incubated with 5 μg of BcSch9-GST and different amounts of BcMkk1-GST as indicated. Proteins associated with the beads or in the total proteins before His pull-down (input) were probed by western blot analysis using the monoclonal mouse anti-His or anti-GST antibodies. (G) Quantification of the binding data in (F). The relative binding activity of BcSch9 with BcRim15^PK^ is calculated as the BcSch9 band intensity at a given concentration of BcMkk1 divided by the BcSch9 band intensity in the absence of BcMkk1. Values on the bars followed by the same letter are not significantly different at *P* = 0.05.

To further verify the relationship between BcMkk1 and BcRim15, the yeast two-hybrid (Y2H) assay was conducted. As shown in Fig 5A, BcRim15 interacted with BcMkk1. In order to examine the potential BcRim15 phosphorylation patterns in ΔBcMkk1, we performed the Phos-tag assay [54]. Unexpectedly, a higher level of phosphorylation of BcRim15^PK^ (the protein kinase domain of BcRim15) was observed in ΔBcMkk1 (Fig 5B and 5C), indicating that BcMkk1 inhibited phosphorylation of BcRim15 in *B*. *cinerea*. Previous studies with the budding yeast have shown that Rim15 could be phosphorylated by PKA, TOR, Sch9 and Pho80-Pho85 kinases and the phosphorylation of Rim15 leads to its inactivation [55,56]. Since PKA, TOR, and Pho80-Pho85 are essential for fungal growth [57,58], we deleted *BcSCH9* in ΔBcMkk1 and found that ΔBcMkk1-BcSch9 exhibited reduced OA production and protease activity compared with ΔBcMkk1 (Fig 5D), indicating that BcMkk1 may hinder the phosphorylation of BcRim15 mediated by BcSch9. To confirm this deduction, first, we determined the interaction of BcRim15 and BcSch9, and found BcRim15 interacted with BcSch9 by the Y2H and *in vitro* binding assays (Fig 5A and 5E). Second, to test the possibility that BcMkk1 may compete with BcSch9 for binding to BcRim15^PK^, we performed a competitive binding assay. Briefly, 5 μg of BcRim15^PK^ was bound to the Ni sepharose beads, then incubated with indicated amounts of BcMkk1 and 5 μg of BcSch9. Subsequently, the protein complex bound to Ni sepharose beads were eluted by SDS buffer after washing with TBS to avoid nonspecific binding, then analyzed by western blotting using the monoclonal mouse anti-GST antibody. As shown in Fig 5F and 5G, the amounts of BcSch9-GST pulled down by BcRim15-His were reduced with increasing amounts of BcMkk1-GST in the mix, which indicating that BcMkk1 competes with BcSch9 for binding to BcRim15^PK^. Third, the phosphorylation level of BcRim15^PK^ in ΔBcSch9 was found to be similar to that in the wild type owing to the presence of BcMkk1, and the phosphorylation level of BcRim15^PK^ in ΔBcMkk1 was reduced by deleting *BcSCH9* (Fig 5C). Taken together, these results strongly indicated that BcMkk1 impeded BcSch9 in phosphorylating BcRim15.

### BcPro40 is a scaffold of the BcBck1-BcMkk1-BcBmp3 cassette

**Fig 6:**
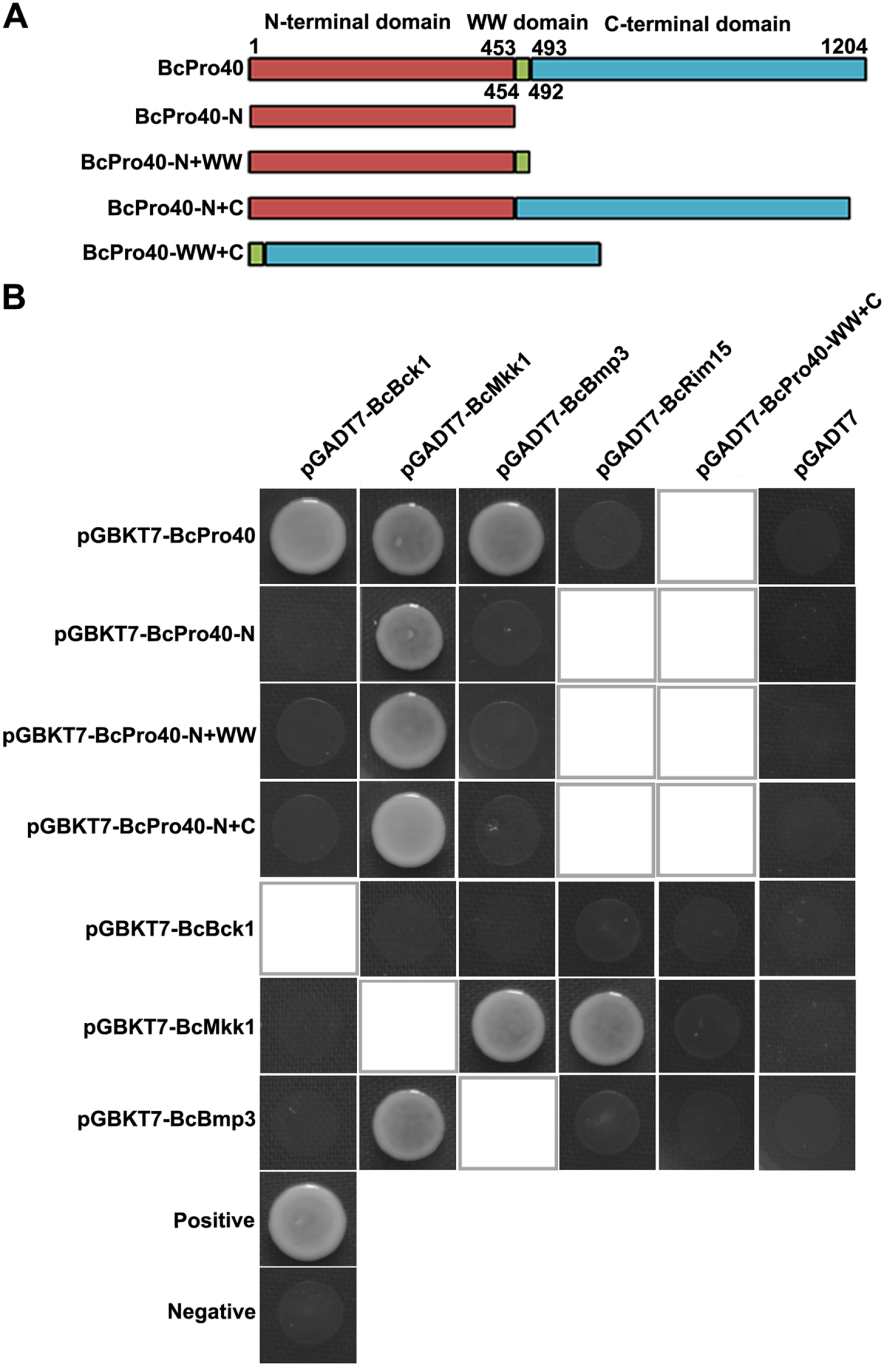
BcPro40 was identified as a scaffold protein for the MAPK cassette of the CWI pathway in *B*. *cinerea*. (A) Structure sketches of BcPro40 used for the yeast two-hybrid analysis. (B) Yeast two-hybrid analysis of interaction of the MAP kinsese of CWI pathway or the PAS kinase BcRim15 with BcPro40 or BcPro40 derivatives. Yeast cells (10^6^ cells ml^–1^) transferred with bait and prey constructs indicated in the figure were drop-plated on SD-Leu-Trp-His plates. A pair of plasmids pGBKT7-53 and pGADT7 was used as a positive control. A pair of plasmids pGBKT7-Lam and pGADT7 was used as a negative control. Empty squares indicate that interactions were not tested.

In *S*. *cerevisiae*, *B*. *cinerea* and *F*. *graminearum*, Ste50 is regarded as the scaffold protein of Ste11-Ste7-Kss1 pathway and plays important roles in vegetative growth or virulence [59–61]. More recently, Pro40 has been identified as the scaffold protein of CWI pathway in filamentous fungus *S*. *macrospora* [14]. Based on the affinity capture assay, two scaffold proteins BcSte50 (Bcin08g03660) and Bcin01g06080 (a homolog of *S*. *macrospora* Pro40, hereafter named BcPro40) were identified as potential BcMkk1-interacting proteins (S2 Table). To clarify the putative scaffolding role of BcSte50 and BcPro40 for the CWI pathway in *B*. *cinerea*, we first determined the sensitivity of mutants to glucanase, OA production and protease activity and using mycelial growth assays. The mutant ΔBcPro40 but not mutant ΔBcSte50 showed increased sensitivity to glucanase, elevated OA production and protease activity (Fig 4D–4F; S4 Fig). Moreover, BcBmp3 phosphorylation was reduced in ΔBcPro40 compared to the wild type after CR treatment (Fig 4F), which is consistent with the elevated sensitivity to cell wall-degrading enzyme glucanex (Fig 4G). The increased OA biosynthesis in ΔBcPro40 was further verified by HPLC and qRT-PCR assays (Fig 4A–4C). Further, we tested the interactions of BcPro40 with three MAPKs of the CWI pathway using the yeast two-hybrid assays. As shown in Fig 6A and 6B, BcPro40 physically interacted with the three MAPKs BcBck1, BcMkk1 and BcBmp3, but not with BcRim15. Further domain analyses showed that the N-terminal domain of BcPro40 was necessary for the interaction with BcMkk1, and that the WW and C-terminal domains were essential for the interaction with BcBck1 and BcBmp3 but dispensable for the interaction with BcMkk1 (Fig 6A and 6B). These results indicated that BcPro40 is a scaffold protein for the MAPK cassette of the CWI pathway in *B*. *cinerea*.

## Discussion

**Fig 7:**
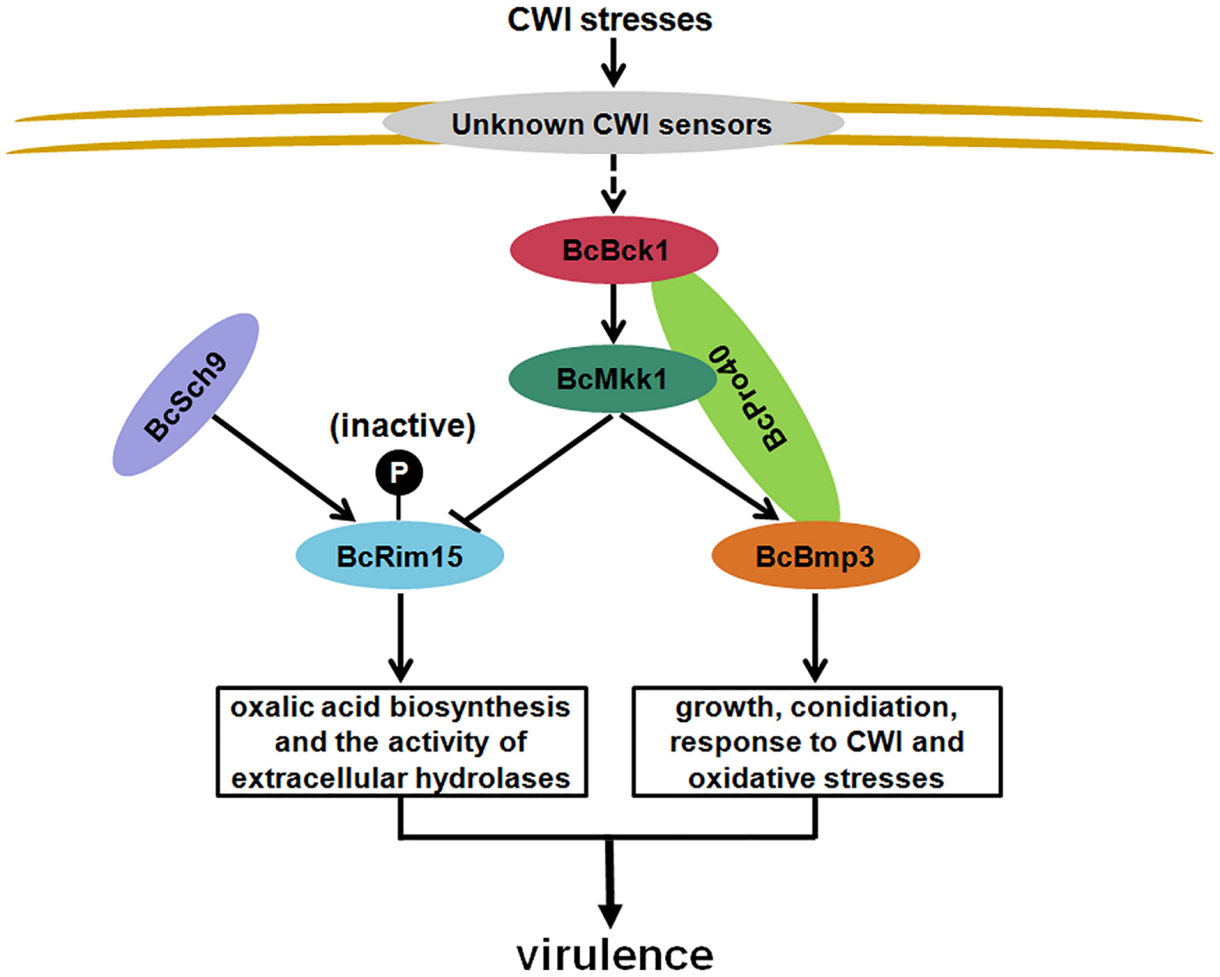
A proposed model for the roles of BcMkk1 in virulence of *B*. *cinerea*. BcMkk1 positively regulates pathogenicity mainly via the cell wall integrity (CWI) pathway, which controls vegetative growth, conidiation and responses to cell wall and oxidative stresses. On the other hand, BcMkk1 impedes phosphorylation of BcRim15 mediated by BcSch9, subsequently inhibits oxalic acid production and the activity of extracellular hydrolases. In addition, BcPro40 is a scaffold protein of the CWI pathway in *B*. *cinerea*. Dotted lines represent unidentified routes.

The conserved CWI pathway is responsible for the integrity of cell wall in many fungal species [11,12,62]. Similar to the reports from other fungi, the MAPK mutants in the CWI pathway of *B*. *cinerea* showed increased sensitivity to CR, which destroys the connection of chitin microfibril to β-glucan [63]. qRT-PCR assays revealed that chitin synthase-encoding genes were dramatically down-regulated in the *B*. *cinerea* MAPK mutants (S5 Fig). Previous studies have shown that the three MAPKs Bck1, Mkk1 and Slt2 function clearly in the same way in *S*. *cerevisiae*, *A*. *fumigatus*, *M*. *oryzae*, *Ustilago maydis*, *Ashbya gossypii* and *S*. *macrospora* [12,14,64–66]. Moreover, the three MAPK mutants of *F*. *graminearum* or *M*. *oryzae* showed similar morphological characters (S6A Fig). However, in this study, ΔBcMkk1 exhibited more serious defects in mycelial growth, conidiation and responses to cell wall and oxidative stresses than ΔBcBck1 and ΔBcBmp3 (Figs 1 and 2), although western blotting and Y2H assays confirmed the three kinases belong to the CWI pathway of *B*. *cinerea* (Figs 2F and 6B). Interestingly, ΔBcMkk1 exhibited higher virulence than ΔBcBck1 and ΔBcBmp3 although deletion of *BcBCK1*, *BcMKK1* or *BcBMP3* led to dramatically decreased virulence on various plants as compared to the wild type. In most pathogenic fungi, the lack of CWI kinases led to decreased fungal virulence [6,9–11,66,67]. However, the negative functions of a MAPK kinase in fungal virulence has not been reported previously. Here, BcMkk1 not only positively regulates virulence resulting from controlling growth, conidiation and the responses to oxidative and cell wall stresses, but negatively modulates virulence via suppressing oxalic acid production and pathogenicity-related hydrolase activity (Fig 7). These results strongly indicated in addition to involvement in the CWI pathway, BcMkk1 carries additional functions in regulating virulence in *B*. *cinerea*.

In *B*. *cinerea*, the VELVET and White Collar complexes were associated with OA formation [68–71], and an activating mutation of transcription factor Pac1 (PacC homolog) gene caused OA increase in *S*. *sclerotiorum* [72]. However, functions of the CWI pathway in regulating OA biosynthesis have not been reported in fungi yet. This study found that BcMkk1, not BcBck1 and BcBmp3 negatively control OA biosynthesis by inhibiting the transcription of *BcOAH*. In contrast, deletion of the BcMkk1 counterpart (MoMkk1) in *M*. *oryzae* led to decreased production of acid(s) (S6A Fig), indicating that MoMkk1 may positively regulate the acid production. Further, we synthesized a BcMkk1 fragment containing 18 mutated amino acids within protein kinase domain that are different from those in *M*. *oryzae* MoMkk1, and found that the mutated BcMkk1 did not interact with BcRim15 anymore (S6B–S6D Fig). These results indicate that Mkk1 negatively modulates OA biosynthesis might be a species-specific trait in *B*. *cinerea*.

Rim15, a PAS family protein kinase, integrates the signals from the TOR, PKA and Pho85-Pho80 kinase (PHO) nutrient sensing pathways, and plays important roles in growth coordination, quiescence entry and lifespan extension in the budding yeast [53,73–75]. More recently, the kinases Rim15 and Sch9 (a downstream effector of TOR) were found to be involved in induction of autophagic degradation of ribosomes in *S*. *cerevisiae* [76], and in oleaginous yeast *Yarrowia lipolytica*, the TORC1-Sch9-Rim15 signaling pathway repressed yeast-to-hypha transition in response to glycerol availability [77]. In this study we found for the first time that BcRim15 negatively regulates production of a secondary metabolite (OA) in *B*. *cinerea*. As expected, ΔBcRim15 displays similar virulence on host tissues compared with the wild type although ΔBcRim15 showed slower growth than the wild type (Fig 4D; S7 Fig), which suggests that the increased acidification ability of ΔBcRim15 contributes to its virulence. Moreover, we found that BcMkk1 interacts with and activates BcRim15 via impeding the phosphorylation of BcRim15 mediated by BcSch9 (Fig 5). In addition, BcRim15 is not required for cell wall integrity (Fig 4F–4H). These results indicate that in addition to BcBmp3, BcRim15 is a second downstream effector of BcMkk1.

Previous studies have found that Rim15 integrates extracellular nutrition signals to downstream effectors including the transcription factors Gis1, Msn2/4 and Hsf1 to regulate starvation-induced stress response in *S*. *cerevisiae* [78,79]. We therefore deleted *BcGIS1* and *BcMSN2/4*, and found that lack of these genes did not change OA production in *B*. *cinerea* (S4 Fig). Given that the transcription factor Rlm1 is regulated by the CWI pathway in many fungi [4,11], deletion of *BcATF1* lead to increased secondary metabolism in *B*. *cinerea* [80], and PacC affects OA formation in *S*. *sclerotiorum* [72], we obtained the deletion mutants of transcription factors BcAtf1, BcRlm1 and BcPacC. Unexpectedly, ΔBcAtf1, ΔBcRlm1 or ΔBcPacC did not show increased OA production (S4 Fig). Thus, more studies are needed to identify transcription factors modulated by BcMkk1 and BcRim15 in regulating OA production.

Enzymes secreted by *B*. *cinerea* that are active in an acidic environment include proteinases [19,46], peroxidases [48] and laccases [19,49]. OA secretion and host tissue acidification that promote hydrolase activity have been suggested to contribute to virulence of *B*. *cinerea* [81]. The *B*. *cinerea* mutants A336 and ΔBcVel1 was impaired in virulence, which is mainly due to the lack of OA secretion and the inability of acidifying host tissue [68,82]. Current study showed that the deletion of *BcMKK1* led to a dramatic increase of OA production, elevated activity of protease, peroxidase and laccase, and higher virulence compared with *BcBCK1* or *BcBMP3* deletion (Fig 3). Moreover, deletion of OA biosynthesis enzyme in ΔBcMkk1 background led to reduced OA production and hydrolase activity and to decreased virulence compared with ΔBcMkk1 (Fig 4D and 4E; S7 Fig). In addition, deletion of *BcOAH* caused reduced laccase activity and limited lesions on some host tissues (Fig 4D and 4E; S7 Fig), as previously reported for ΔBcOah by Liu et al. and Müller et al. [31,32]. Taken together, these results confirm that OA secretion contributes to *B*. *cinerea* virulence via acidifying host tissues and subsequently enhancing hydrolase activity.

Scaffold proteins are a class of signaling organizers that play vital roles not only in assembling signaling complexes by binding at least two signaling proteins together, but also in regulating signaling outputs by fine-tuning the crosstalk among the proteins within the complex [83,84]. In the budding yeast, the polarisome component Spa2p is found to perform as a scaffold-like protein for the CWI pathway during polar growth [85]. However information about scaffold proteins of the CWI pathway is very limited in filamentous fungi. In this study, our data revealed that BcPro40 acts as a scaffold protein for the CWI pathway in *B*. *cinerea*. Teichert *et al*. [14] also reported that Pro40 served as a scaffold protein for CWI pathway in *S*. *macrospora*, but current studies showed that the organization modes of signal complexes and functions of scaffold Pro40s are largely different in *B*. *cinerea* and *S*. *macrospora*. First, SmPro40 interacted with SmMik1 (the Bck1 ortholog) and SmMek1 (the Mkk1/2 ortholog) but not with SmMak1 (the Slt2 homolog), whereas BcPro40 could interact with all the three components of CWI pathway in *B*. *cinerea*. Moreover, the N-terminal domain of Pro40 is required for the interaction of BcPro40 with BcMkk1, but not for the interaction of SmPro40 with SmMek1. Additionally, the whole Pro40 is necessary for the interaction of Pro40 with the MAPKKK in *B*. *cinerea* (BcBck1) but not in *S*. *macrospora* [14]. Finally, the Pro40 ortholog (SmPro40) plays roles in sexual development and hyphal fusion, but not in cell wall integrity in *S*. *macrospora* [14]. In contrast, BcPro40 is involved in cell wall integrity, the regulation of OA biosynthesis and virulence in *B*. *cinerea* (Fig 4; S7 Fig).

In conclusion, we have found that in addition to its involvement in the CWI pathway, the MAPK kinase (BcMkk1) negatively regulates OA biosynthesis, and the BcMkk1-interacting protein BcRim15 is an effector of BcMkk1 in negative regulation of OA production. Furthermore, BcPro40 was identified as a scaffold protein for the CWI pathway in *B*. *cinerea*. Collectively, our findings provide important insights into the conserved and species-special functions of the CWI pathway in the pathogenic fungus *B*. *cinerea*.

## Materials and methods

### Fungal strain and growth assays

*B*. *cinerea* strain 38B1 (CGMCC 4006) isolated from grape in California, USA was used as the wild-type strain for constructing gene deletion mutants. The wild-type strain, resultant gene deletion and complemented strains were cultured at 25°C on PDA (200 g potato, 20 g dextrose, 20 g agar, and 1 l water) amended with CR, glucanase, paraquat or H_2_O_2_ at concentrations as indicated in the figure legends for mycelial growth assays. Each plate was inoculated with a 5 mm diameter mycelial plug taken from the edge of a 3-day-old colony grown on PDA. After the incubation at 25°C for 3 days, the percentage of relative mycelial growth was calculated. Additionally, each strain was grown on PDA for 10 days to count the conidium number. Each experiment was repeated three times.

### Construction of gene deletion and complementation mutants

The double-joint PCR approach was used to generate the gene replacement construct for each target gene [86]. In briefly, the 5’ and 3’ flanking regions of each gene were amplified with the primer pairs listed in S1 Table, and the amplified sequences were then fused with the hygromycin resistance gene cassette (*HPH*). The resulting PCR products for each gene were transformed into protoplasts of the wild-type progenitor 38B1 respectively, as described previously [87]. Hygromycin B (Calbiochem, La Jolla, CA) was added to a final concentration of 100 mg l^–1^ for transformant selection. Putative gene deletion mutants were identified by PCR assays, and were further analyzed by the Southern blotting assay.

To construct of BcMkk1-GFP fusion cassette, the open reading frame of BcMkk1 was amplified and assembled with the NotI-digested pNAN-OGG [88] using a One Step Cloning Kit (Vazyme biotech, Nanjing, China). The resulting recombinational plasmids were verified by sequencing to ensure the accuracy of the in-frame fusion region. For transformation, the sequenced vector was cut with SacII and ApaI to obtain the linearized BcMkk1-GFP fusion constructs. Using the similar strategy, the BcBck1-, BcBmp3-, and BcRim15 protein kinase domain (BcRim15^PK^)-GFP fusion cassettes were also constructed. Nourseothricin (Beijing Solarbio Science and Technology Co., Ltd.) was added to a final concentration of 50 mg l^–1^ for transformant selection. The resulting complemented strains were identified by PCR assays, and examined for GFP signals with the Zeiss LSM780 confocal microscope (Carl Zeiss AG, Germany). All of the mutants generated in this study were preserved in 15% glycerol at −80°C.

### Determination of ROS quantity

To measure ROS content, fresh mycelia of the wild type and the deletion mutants grown in PDB were harvested, washed with deionized water. About 50 mg of finely ground mycelia were resuspended in 1 ml of extraction buffer S0038-3 (Beyotime Industrial Co., Ltd., Shanghai, China). After homogenization with a vortex shaker, the lysate was centrifuged at 14 000 *g* in a microcentrifuge for 10 min at 4°C. An aliquot of 10 μl supernatant was used for ROS content determination with ROS ELISA Kit (Tong Wei Industrial Co., Ltd., Shanghai, China). The experiment was repeated three times.

### Pathogenicity and infection-related morphogenesis assays

Leaves of 4-week-old tomato plants, fruits of strawberry, pear and apple were inoculated with 5 mm diameter plugs of 3-day-old cultures. Ten leaves and ten strawberry, pear or apple fruits were used for each strain. Before inoculation, leaves and fruits were wounded with a sterilized needle to facilitate penetration of the fungus into plant tissue. Inoculated tissues were incubated at 25°C with 16 h of daylight. Disease lesions were photographed 60 h after inoculation. The experiment was repeated four times.

Infection-related morphogenesis was observed on onion epidermis as previously described [89]. Mycelial plugs from 3-day-old cultures were inoculated on the hydrophobic side of the epidermis. After 24 h or 36 h of incubation in a humid environment at 25°C, the epidermis was stained with aniline blue before microscopic evaluation [90]. Fungal mycelia were observed under light transmission microscopy.

### Extracellular hydrolase activity and organic acids detection assays

Each strain was cultured in liquid complete medium (CM; 1% glucose, 0.2% peptone, 0.1% yeast extract, 0.1% casamino acids, nitrate salts, and trace elements, and 0.01% vitamins, pH 6.5), and mycelia were removed completely by filtration and centrifugation (5000 *g* at 4°C) after 5 days. Laccase and peroxidase activities were measured using a colorimetric determination as described previously [91]. Protease activity was determined by the Protease ELISA Kit (Tong Wei Industrial Co., Ltd., Shanghai, China).

The pH-indicating medium employed 0.05% bromothymol blue as indicator that was amended to PDA plate, and the medium color changed from green to yellow denoting the production of acid compounds [71]. For detection of the secretion of citric, malic, succinic, oxalic and fumaric acids, 100 ml of PDB in 250-ml flasks were inoculated with 4 mycelial plugs (5 mm in diameter) from the edge of 3-day-old cultures, incubated at 25°C in 150 rpm shaker for 5 days, then about 1 ml supernatant was filtrated with sterile Millex Filter Units (0.22 μm) for use. Enzymatic assay kits were provided by Tong Wei Industrial Co., Ltd. (Shanghai, China) and assays were performed according to the manufacturer’s instructions. For oxalic acid quantification, HPLC was further performed using an Agilent 1100 series system with an Agilent ZORBAX SB-C18 column (4.6 by 250 mm) using 10% methanol as mobile phase, and ultraviolent detection at 210 nm. These experiments were repeated three times.

### Western blotting assay

Fresh mycelia (200 mg) of each strain were finely ground and suspended in 1 ml of extraction buffer (50 mM Tris-HCl, pH7.5, 100 mM NaCl, 5 mM EDTA, 1% Triton X-100, 2 mM PMSF) and 10 μl of protease inhibitor cocktail (Sangon Co., Shanghai, China). After homogenization with a vortex shaker, the lysate was centrifuged at 10 000 *g* in a microcentrifuge for 20 min at 4°C. The resulting proteins were separated on 10% denaturating polyacrylamide gel (SDS-PAGE) and transferred to Immobilon-P transfer membrane (Millipore, Billerica, MA, USA) with a Bio-Rad electroblotting apparatus. The monoclonal anti-GFP ab32146 (Abcam, Cambridge, MA, USA) antibody was used at a 1:5000 to 1:10000 dilution for BcMkk1-GFP immunoblot assays. The phosphorylated BcBmp1 and BcBmp3 were detected with phospho-p44/42 MAPK antibody (Cell Signaling Technology, Boston, MA, USA). The total BcBmp1 and BcBmp3 were detected using p44/42 MAPK antibody (Cell Signaling Technology Inc., Beverly, MA, USA) and anti-Mpk1 antibody (Santa Cruz Biotechnology, Santa Cruz, CA, USA), respectively. Incubation with a secondary antibody and chemiluminescent detection were performed as described previously [92]. The experiment was conducted three times independently.

### Affinity capture-mass spectrometry analysis

BcMkk1 was tagged with GFP and transferred in the *BcMKK1* deletion mutant, and the resulting transformant was used for protein extraction as described above. After centrifugation at 10 000 *g* for 20 min at 4°C, supernatant (800 μl) was transferred into a sterilized eppendorf tube. About 50 μl of GFP-trap agarose beads (ChromoTek, Martinsried, Germany) was added to capture BcMkk1-GFP interacting proteins, following the manufacturer’s instructions. After incubation at 4°C overnight, the agarose was washed three times with 500 μl of TBS (20 mM Tris-HCl, 500 mM NaCl, pH7.5). Proteins binding to the beads were boiled with 60 μl TBS supplemented with 10 μl 10% SDS. After centrifugation at 5000 *g* for 5 min at 4°C, supernatant was digested with trypsin as described previously [93,94]. Tryptic peptides were analyzed by mass spectrometry using a previous published protocol [95].

### Yeast two-hybrid assays

To construct plasmids for yeast two-hybrid analyses, the coding sequence of each tested gene was amplified from the cDNA of 38B1 with primer pairs indicated in S1 Table. The cDNA of each gene was inserted into the yeast GAL4 binding domain vector pGBKT7 and GAL4 activation domain vector pGADT7 (Clontech, Mountain View, CA, USA), respectively. The pairs of yeast two-hybrid plasmids were co-transformed into *S*. *cerevisiae* strain AH109 following the LiAc/SS-DNA/PEG transformation protocol [96]. In addition, a pair of plasmids pGBKT7-53 and pGADT7 served as a positive control. A pair of plasmids pGBKT7-Lam and pGADT7 was used as a negative control. Transformants were grown at 30°C for 3 days on synthetic medium (SD) lacking Leu and Trp, and then transferred to SD stripped of His, Leu and Trp and containing 5 mM 3-aminotriazole (3-AT) to assess binding activity [97]. Three independent experiments were performed to confirm yeast two-hybrid assay results.

### Phos-tag analysis

The BcRim15^PK^-GFP fusion construct was transferred into the wild-type strain and ΔBcMkk1 respectively. The resulting transformants were used for protein extraction as described above. The proteins were separated on 8% SDS-polyacrylamide gels prepared with 25 μM Phos binding reagent acrylamide (APExBIO, F4002) and 100 μM ZnCl_2_. Gels were electrophoresed at 20 mA gel^–1^ for 3–5 h. Prior to transfer, gels were first equilibrated in transfer buffer containing 5 mM EDTA for 5 min three times and then in transfer buffer without EDTA for 5 min two times. Protein transfer from the Zn^2+^-phos-tag acrylamide gel to the PVDF membrane was performed 4–5 h at 100 V at 0°C, and then the membrane was analyzed by western blotting with anti-GFP antibody.

### *In vitro* protein-binding assay

cDNAs encoding full-length BcSch9 and BcMkk1 were amplified by PCR and subcloned into pGEX-4T-3 to generate corresponding GST fusion proteins. cDNAs encoding BcRim15^PK^ were amplified and cloned into pET-22b(+) to generate His-tagged proteins. Then *Escherichia coli* strain BL21 (Invitrogen, Carlsbad, CA) was transformed with above constructs, grown in 1000 ml LB medium containing 100 μg ml^–1^ ampicillin at 37°C. After the OD_600_ had reached ∼0.5, IPTG was added to a final concentration of 0.5 mM and the cultures were incubated at 16°C. After 8 h incubation, *E*. *coli* strain BL21 cells were collected by centrifugation at 4°C and suspended in TBS buffer containing 1% Triton X-100 (final concentration) and proteinase inhibitor cocktail. Protein extracts were obtained by sonicated cells for 9 x 10 s with 10 s interval on ice, centrifuged at 7200 *g* for 20 min at 4°C and recombinant proteins were then purified from protein extracts according to corresponding manufacturer’s instructions. The concentrations of the purified recombinant proteins were estimated by comparing the sample proteins to bovine serum albumin (BSA) of known concentrations by SDS-PAGE analysis, followed by staining the gel with coomassie brilliant blue.

To test *in vitro* binding between His- and GST-tagged proteins, 3 μg of GST-tagged protein or GST (negative control) that was still bound to the glutathione beads was mixed with 10 μg of His-tagged protein and rocked for 2 h at 4°C. The beads were washed six times with cold TBS and resuspended in 5 x SDS sample buffer, and GST pull-down proteins were then analyzed by western blotting using monoclonal mouse anti-His antibody ab18184 (Abcam, Cambridge, MA, USA). The experiment was conducted two times independently.

For competitive *in vitro* binding assay, indicated amounts of BcMkk1 mixed with 5 μg of BcSch9 were incubated with 5 μg of BcRim15^PK^ bound to the Ni sepharose beads for 2 h. The protein complex bound to Ni sepharose beads were eluted by 5 x SDS buffer after washing six times with 1 x TBS to avoid nonspecific binding, boiled for 5 min, then analyzed by western blotting using the monoclonal mouse anti-GST antibody M0807-1 (Hangzhou HuaAn Biotechnology Co., Ltd.). The experiment was conducted two times independently.

## Supporting information

S1 Fig *B*. *cinerea* mitogen-activated protein kinases (MAPKs) in the cell wall integrity (CWI) pathway are homologous to those counterparts from yeasts and other filamentous fungi. Phylogenetic tree generated using the neighbor-joining method with Mega 5.0 software on the basis of the deduced amino acid sequences of MAPK orthologs from different fungi. BcBck1 (GenBank accession no. XP_001550572.1), BcMkk1 (XP_001549887.1) and BcBmp3 (XP_001554555.1) from *B*. *cinerea* that were indicated with black dots; ScBck1 (NP_012440.1), ScMkk1 (NP_014874.1), ScMkk2 (NP_015185.1) and ScSlt2 (NP_011895.1) from *Saccharomyces cerevisiae*; AnBck1 (CBF76548.1), AnMkk1 (XP_661793.1) and AnMpk1 (AAD24428.1) from *Aspergillus nidulans*; FgBck1 (XP_011324981.1), FgMkk1 (XP_011327039.1), and FgMgv1 (XP_011319273.1) from *Fusarium graminearum*; MoMck1 (ELQ43863.1), MoMkk1 (ELQ59117.1) and MoMps1 (XP_003712437.1) from *Magnaphorthe oryzae*; NcBck1 (XP_011395111.1), NcMkk1 (XP_957310.3) and NcMpk1 (XP_958040.2) from *Neurospora crassa*; UmBck1 (XP_011387646.1), UmMkk1 (XP_011391763.1) and UmMpk1 (XP_011386664.1) from *Ustilago maydis*; and PgBck1 (XP_003328672.2), PgMkk1 (XP_003890718.1) and PgMpk1 (XP_003335205.1) from *Puccinia graminis*. The bootstrap values are indicated on the phylogenetic tree.
(TIF)

S2 Fig Schematic representation of the *BcBCK1*, *BcMKK1*, *BcBMP3*, *BcOAH*, *BcRIM15* and *BcPRO40* disruption strategy. (A) Each gene and hygromycin resistance cassette [HPH] are denoted by large black and gray arrows, respectively. (B) PCR assays for identification of gene deletion mutants and the complemented strains ΔBcBck1-C, ΔBcMkk1-C and ΔBcBmp3-C. (C) Southern blotting analyses of the deletion mutants. A 875-bp fragment of *HPH* gene was used as the probe in the Southern blotting assays. The restriction enzyme used for digestion of genomic DNA preparation is indicated in schematic representation of the disruption strategy for each strain. The size of the resulting hybridization band is indicated for each strain at the bottom of Southern blotting image.
(TIF)

S3 Fig Subcellular localization of BcMkk1-GFP in *B*. *cinerea*. The strain ΔBcMkk1::BcMkk1-GFP was stained with DAPI (4',6-diamidino-2-phenylindole, a nucleus tracker) or CMAC (7-amino-4-chloromethylcoumarin, a vacuole tracker). Scale bar = 10 μm.
(TIF)

S4 Fig Analyses of acid production, protease activity and sensitivity to cell wall-damaging agent glucanase for BcMkk1-interacting protein mutants (ΔBcSte20, ΔBcHog1, ΔBcSch9 and ΔBcSte50) and transcription factors possibly downstream of BcRim15 mutants (ΔBcAtf1, ΔBcRlm1, ΔBcGis1, ΔBcMsn2/4 and ΔBcPacC). Each strain was inoculated on PDA amended with 0.05% bromothymol blue indicating acid production, skimmed milk agar medium for protease detection, and PDA supplemented with 22.5 mg ml^–1^ glucanase for checking the cell wall integrity, then incubate at 25°C for 3 or 5 days.
(TIF)

S5 Fig Effect of *BcBCK1*, *BcMKK1* or *BcBMP3* deletion on the transcription of eight *BcCHS* genes assayed by qRT-PCR. The relative expression level of each *BcCHS* gene in each deletion mutant is the relative amount of mRNA in the wild type. Line bars in each column denote standard errors of three repeated experiments. Values on the bars followed by the same letter for each *BcCHS* gene are not significantly different at *P* = 0.05.
(TIF)

S6 Fig The function of Mkk1 in regulating acid production is different between *Botrytis cinerea* and *Magnaporthe oryzae*. (A) The MAPK mutants of the CWI pathway positively modulate acid production in *M*. *oryzae*. The MAPK mutants as well as the wild type of *Fusarium graminearum* displayed undetectable acid production. The wild-type strain Guy11 and PH-1, and the deletion mutants ΔMoMck1, ΔMoMkk1, ΔMoMps1, ΔFgBck1, ΔFgMkk1 and ΔFgMgv1 were cultured on PDA with or without acid-indicating agent (0.05% bromothymol blue) at 25°C for 3 days. (B) Mkk1 orthologs from *B*. *cinerea* and *M*. *oryzae* showed one conserved protein kinase domain identified by Pfam (http://pfam.xfam.org/). (C) Alignments of amino acid sequences of protein kinase domain of BcMkk1 and MoMkk1 were performed on http://tcoffee.crg.cat/apps/tcoffee/do:mcoffee. Identical (red shading) or similar (yellow shading) amino acids were highlighted. (D) The mutated BcMkk1 containing 18 point mutations within the protein kinase domain could not interact with BcRim15. Serial concentrations of yeast cells were drop-plated on SD-Leu-Trp-His plates. A pair of plasmids pGBKT7-53 and pGADT7 was used as a positive control. A pair of plasmids pGBKT7-Lam and pGADT7 was used as a negative control.
(TIF)

S7 Fig Pathogenicity assays on different plant tissues, following inoculation with the wild-type strain 38B1, ΔBcOah, ΔBcMkk1, ΔBcMkk1-BcOah, ΔBcRim15 and ΔBcPro40. Agar plugs without fungal mycelia were used as negative controls (NCKs). Disease symptoms were photographed and diameter of disease lesions were measured 60 h post inoculation (hpi). Values on the bars followed by the same letter are not significantly different at *P* = 0.05.
(TIF)

S1 Table Oligonucleotide primers used in this study and their relevant characteristics. (DOCX)

S2 Table A list of partial BcMkk1-interacting proteins identified by affinity capture in coupling with mass spectrometry. (DOCX)
