# Supplementary figures and images for: The MAPK kinase BcMkk1 suppresses oxalic acid biosynthesis via impeding phosphorylation of BcRim15 by BcSch9 in Botrytis cinerea
Source: PLoS Pathog. 2018 Sep 13;14(9):e1007285. doi: 10.1371/journal.ppat.1007285 (PMC6136818; doi:10.1371/journal.ppat.1007285)

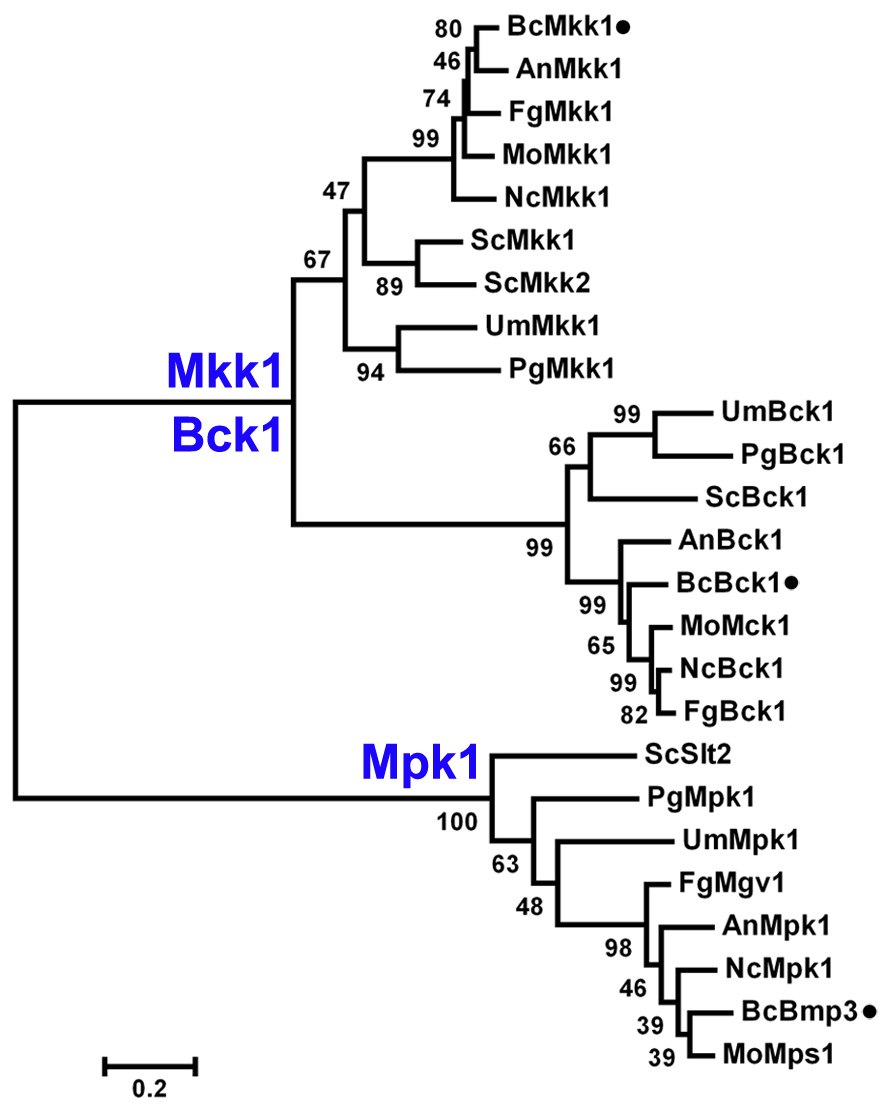

Supplement: S1 Fig — Phylogenetic tree generated using the neighbor-joining method with Mega 5.0 software on the basis of the deduced amino acid sequences of MAPK orthologs from different fungi. BcBck1 (GenBank accession no. XP_001550572.1), BcMkk1 (XP_001549887.1) and BcBmp3 (XP_001554555.1) from B. cinerea that were indicated with black dots; ScBck1 (NP_012440.1), ScMkk1 (NP_014874.1), ScMkk2 (NP_015185.1) and ScSlt2 (NP_011895.1) from Saccharomyces cerevisiae; AnBck1 (CBF76548.1), AnMkk1 (XP_661793.1) and AnMpk1 (AAD24428.1) from Aspergillus nidulans; FgBck1 (XP_011324981.1), FgMkk1 (XP_011327039.1), and FgMgv1 (XP_011319273.1) from Fusarium graminearum; MoMck1 (ELQ43863.1), MoMkk1 (ELQ59117.1) and MoMps1 (XP_003712437.1) from Magnaphorthe oryzae; NcBck1 (XP_011395111.1), NcMkk1 (XP_957310.3) and NcMpk1 (XP_958040.2) from Neurospora crassa; UmBck1 (XP_011387646.1), UmMkk1 (XP_011391763.1) and UmMpk1 (XP_011386664.1) from Ustilago maydis; and PgBck1 (XP_003328672.2), PgMkk1 (XP_003890718.1) and PgMpk1 (XP_003335205.1) from Puccinia graminis. The bootstrap values are indicated on the phylogenetic tree. (TIF) [file ppat.1007285.s001.tif]

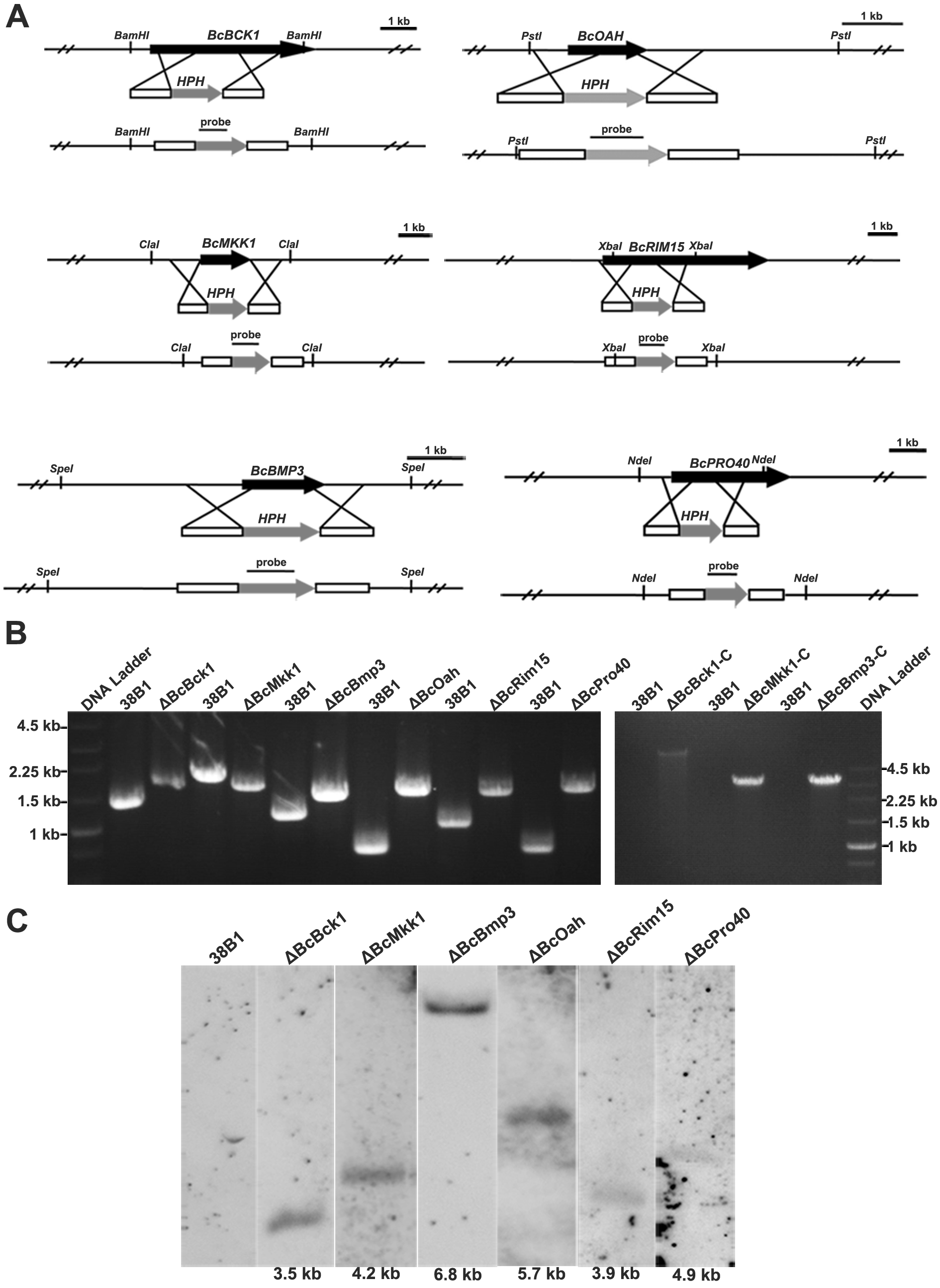

Supplement: S2 Fig — (A) Each gene and hygromycin resistance cassette [HPH] are denoted by large black and gray arrows, respectively. (B) PCR assays for identification of gene deletion mutants and the complemented strains ΔBcBck1-C, ΔBcMkk1-C and ΔBcBmp3-C. (C) Southern blotting analyses of the deletion mutants. A 875-bp fragment of HPH gene was used as the probe in the Southern blotting assays. The restriction enzyme used for digestion of genomic DNA preparation is indicated in schematic representation of the disruption strategy for each strain. The size of the resulting hybridization band is indicated for each strain at the bottom of Southern blotting image. (TIF) [file ppat.1007285.s002.tif]

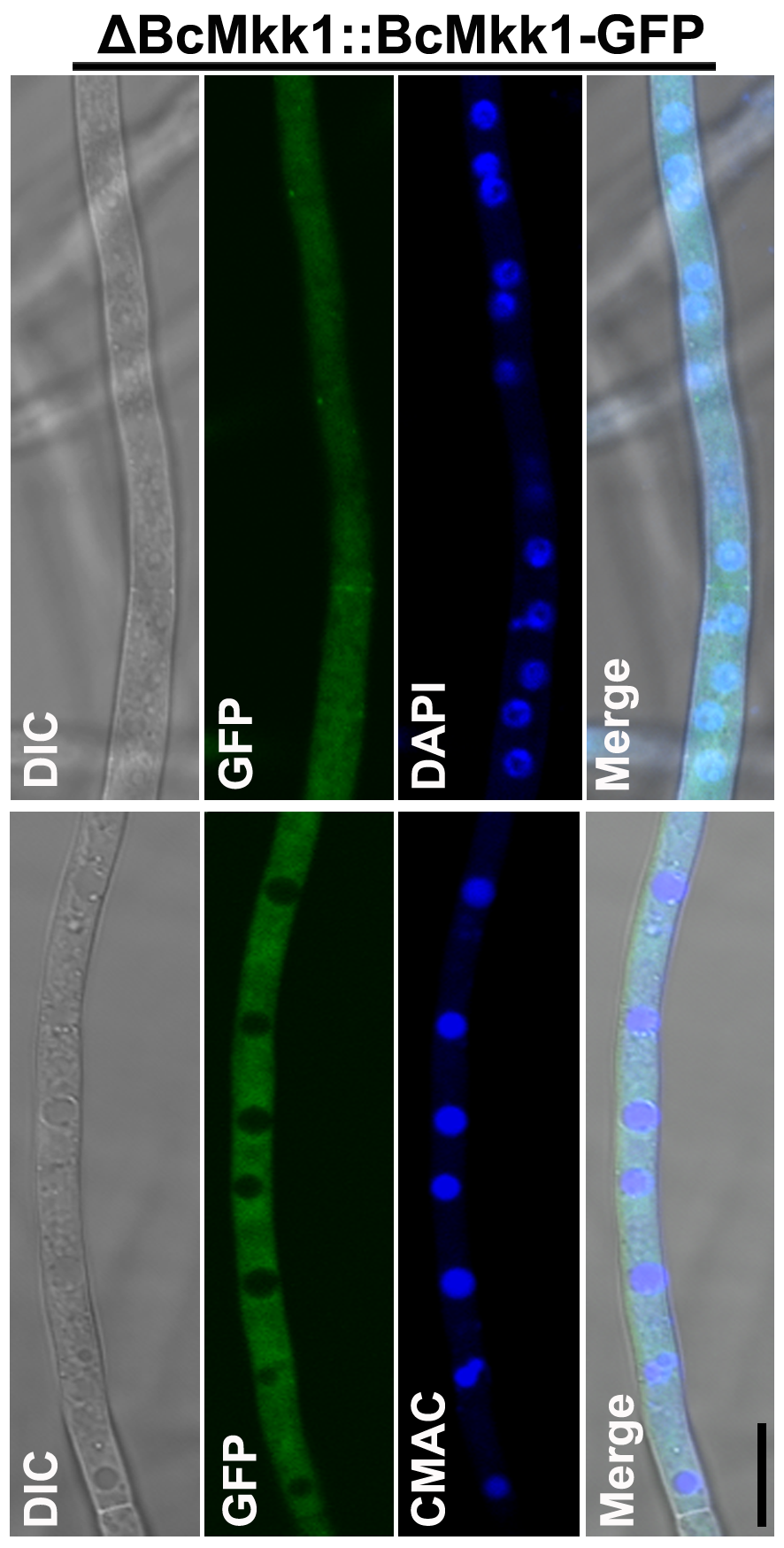

Supplement: S3 Fig — The strain ΔBcMkk1::BcMkk1-GFP was stained with DAPI (4',6-diamidino-2-phenylindole, a nucleus tracker) or CMAC (7-amino-4-chloromethylcoumarin, a vacuole tracker). Scale bar = 10 μm. (TIF) [file ppat.1007285.s003.tif]

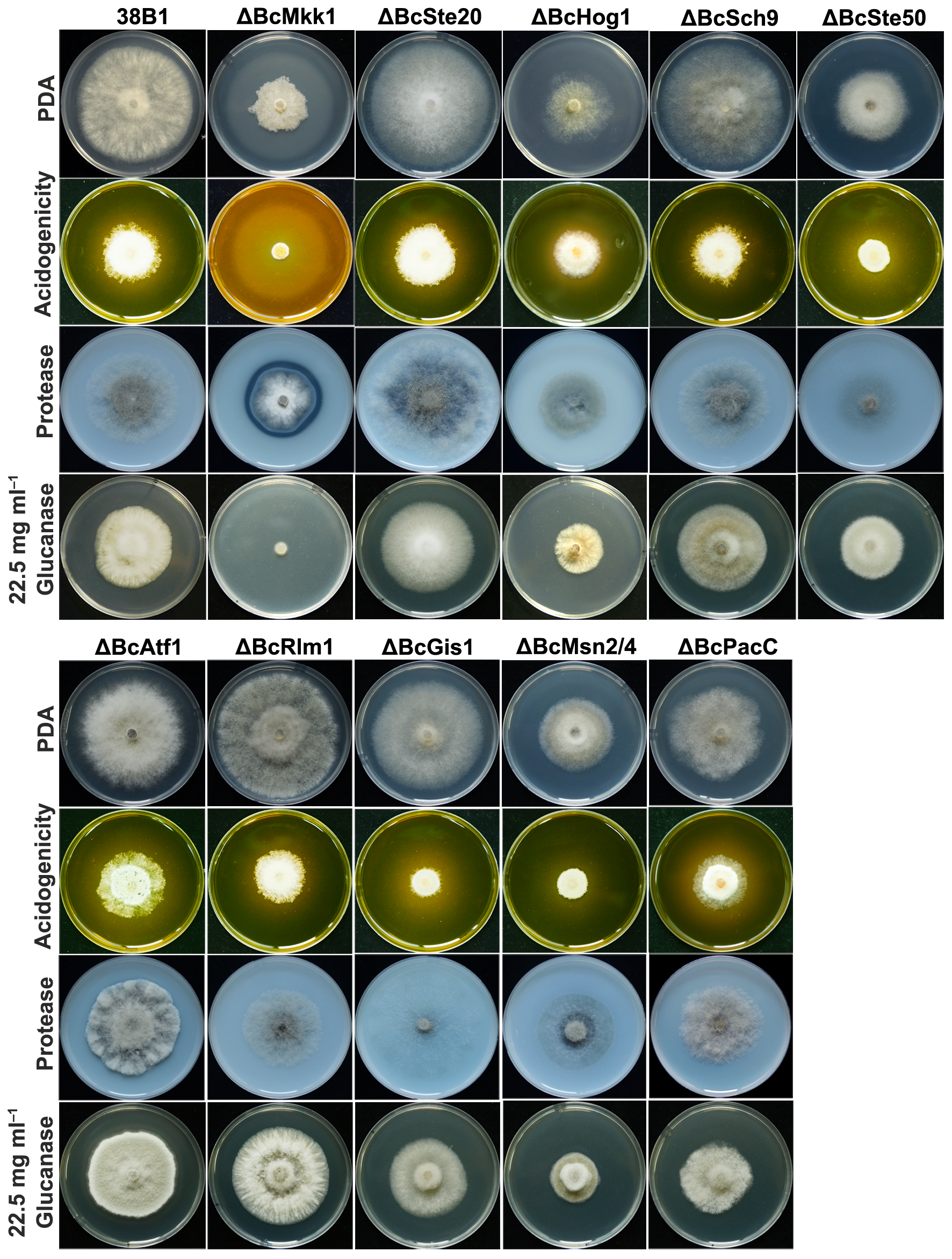

Supplement: S4 Fig — Each strain was inoculated on PDA amended with 0.05% bromothymol blue indicating acid production, skimmed milk agar medium for protease detection, and PDA supplemented with 22.5 mg ml–1 glucanase for checking the cell wall integrity, then incubate at 25°C for 3 or 5 days. (TIF) [file ppat.1007285.s004.tif]

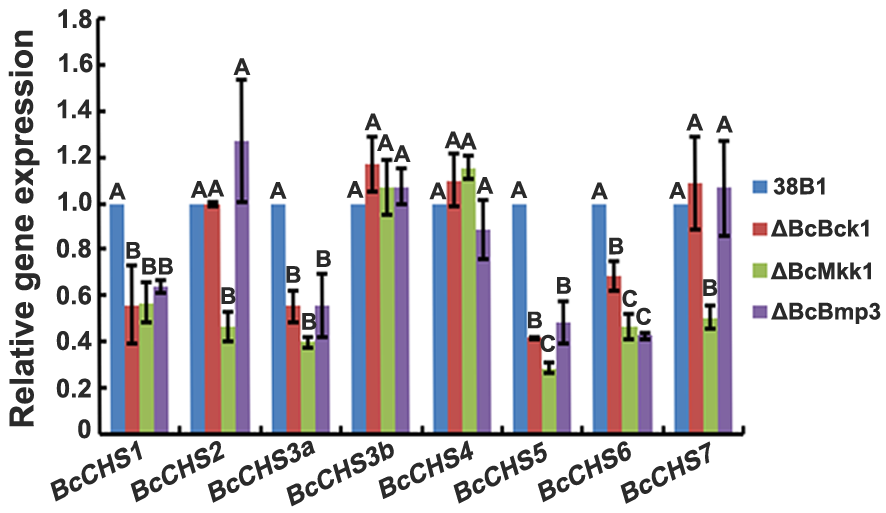

Supplement: S5 Fig — The relative expression level of each BcCHS gene in each deletion mutant is the relative amount of mRNA in the wild type. Line bars in each column denote standard errors of three repeated experiments. Values on the bars followed by the same letter for each BcCHS gene are not significantly different at P = 0.05. (TIF) [file ppat.1007285.s005.tif]

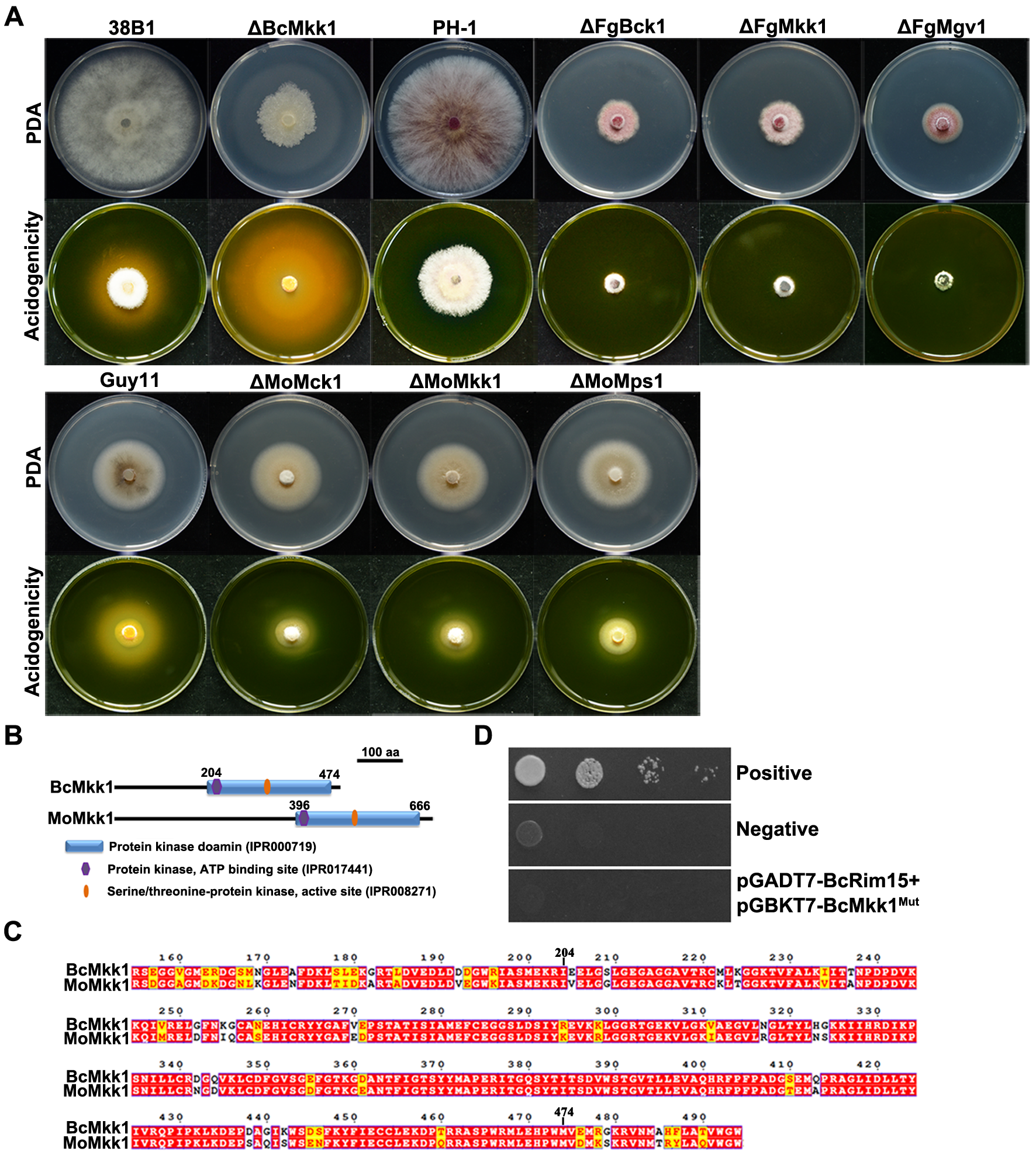

Supplement: S6 Fig — (A) The MAPK mutants of the CWI pathway positively modulate acid production in M. oryzae. The MAPK mutants as well as the wild type of Fusarium graminearum displayed undetectable acid production. The wild-type strain Guy11 and PH-1, and the deletion mutants ΔMoMck1, ΔMoMkk1, ΔMoMps1, ΔFgBck1, ΔFgMkk1 and ΔFgMgv1 were cultured on PDA with or without acid-indicating agent (0.05% bromothymol blue) at 25°C for 3 days. (B) Mkk1 orthologs from B. cinerea and M. oryzae showed one conserved protein kinase domain identified by Pfam (http://pfam.xfam.org/). (C) Alignments of amino acid sequences of protein kinase domain of BcMkk1 and MoMkk1 were performed on http://tcoffee.crg.cat/apps/tcoffee/do:mcoffee. Identical (red shading) or similar (yellow shading) amino acids were highlighted. (D) The mutated BcMkk1 containing 18 point mutations within the protein kinase domain could not interact with BcRim15. Serial concentrations of yeast cells were drop-plated on SD-Leu-Trp-His plates. A pair of plasmids pGBKT7-53 and pGADT7 was used as a positive control. A pair of plasmids pGBKT7-Lam and pGADT7 was used as a negative control. (TIF) [file ppat.1007285.s006.tif]

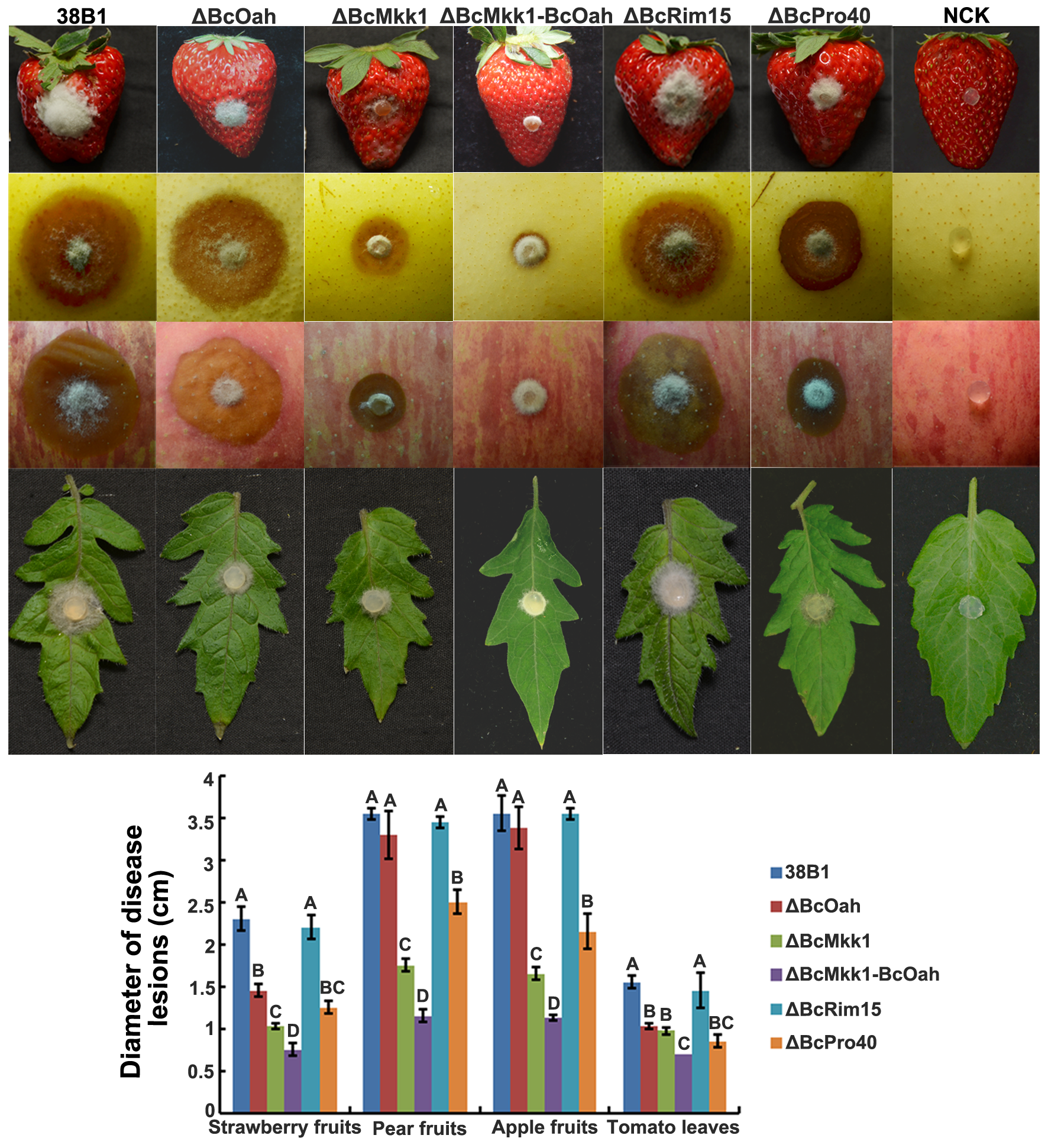

Supplement: S7 Fig — Agar plugs without fungal mycelia were used as negative controls (NCKs). Disease symptoms were photographed and diameter of disease lesions were measured 60 h post inoculation (hpi). Values on the bars followed by the same letter are not significantly different at P = 0.05. (TIF) [file ppat.1007285.s007.tif]
