# Supplementary material for: The MAPK kinase BcMkk1 suppresses oxalic acid biosynthesis via impeding phosphorylation of BcRim15 by BcSch9 in Botrytis cinerea
Source: PLoS Pathog. 2018 Sep 13;14(9):e1007285. doi: 10.1371/journal.ppat.1007285 (PMC6136818; doi:10.1371/journal.ppat.1007285)
Supplement: S1 Table — (DOCX) [file ppat.1007285.s008.docx]

**Table S1** Oligonucleotide primers used in this study and their relevant characteristics.

| Primer | Sequence (5’-3’) | Relevant Characteristics |
| --- | --- | --- |
| P1 | TCCACGGCTAGCAAAAGAGT | Primers to amplify *BcBCK1* upstream fragment for construction of the gene deletion vector |
| P2 | CAAAATAGGCATTGATGTGTTGACCTCCCCCTTTCCATGAGC |  |
|  |  |  |
| P3 | CTCGTCCGAGGGCAAAGGAATAGAGTAGCAAAAATGCCATGTCGACAG | Primers to amplify *BcBCK1* downstream fragment for construction of the gene deletion vector |
| P4 | AACGCTTATTTGCTTCGTGG |  |
|  |  |  |
| P5 | ACGACCAGAATCTTGATTCCC | PCR primers for identification of *BcBCK1* deletion transformants |
| P6 | TCACCTCTGCCCCAAGTAACA |  |
|  |  |  |
| P7 | AAACTCTTGTCGTACGCGCAT | Primers to amplify *BcMKK1* upstream fragment for construction of the gene deletion vector |
| P8 | CAAAATAGGCATTGATGTGTTGACCTCCTGTTGGGTAATGGGTTGAGA |  |
|  |  |  |
| P9 | CTCGTCCGAGGGCAAAGGAATAGAGTAGTATGGGGCTGGTGATCTGACT | Primers to amplify *BcMKK1* downstream fragment for construction of the gene deletion vector |
| P10 | CTCGGGTTGATTCTGAAATG |  |
|  |  |  |
| P11 | TGACCCGAGATTCGGAACT | PCR primers for identification of *BcMKK1* deletion transformants |
| P12 | AAAATCGAGGAGCGGATCTT |  |
|  |  |  |
| P13 | TGAAACATTCAGCCTTATCCA | Primers to amplify *BcBMP3* upstream fragment for construction of the gene deletion vector |
| P14 | CAAAATAGGCATTGATGTGTTGACCTCCTCGGAAGGAAAGTAACATCGT |  |
|  |  |  |
| P15 | CTCGTCCGAGGGCAAAGGAATAGAGTAGTTGATCCATCTTCCCGTATCG | Primers to amplify *BcBMP3* downstream fragment for construction of the gene deletion vector |
| P16 | AAAATCCAGCTTGCGCCTTA |  |
|  |  |  |
| P17 | GGTCTTCAAGGTCTTCAACCA | PCR primers for identification of *BcBMP3* deletion transformants |
| P18 | AAATGTTGTTGGGCATCCAG |  |
|  |  |  |
| P19 | GGCATTTCTTTGTATGGCAGG | Primers to amplify *BcOAH* uptream fragment for construction of the gene deletion vector |
| P20 | CAAAATAGGCATTGATGTGTTGACCTCCCAAGCGGGAAGCAGTGGTA |  |
|  |  |  |
| P21 | CTCGTCCGAGGGCAAAGGAATAGAGTAGATGGCCGCAAAGACTGTT | Primers to amplify *BcOAH* downstream fragment for construction of the gene deletion vector |
| P22 | TTCATCAGGTGCCACTGCATA |  |
|  |  |  |
| P23 | GTTTGCCCTGGTGTTTATGA | PCR primers for identification of *BcOAH* deletion transformants |
| P24 | TACGGAATCCCATCTCTTGTG |  |
|  |  |  |
| P25 | AAGTCTAAGGGCACCATTCAA | Primers to amplify *BcRIM15* upstream fragment for construction of the gene deletion vector |
| P26 | CAAAATAGGCATTGATGTGTTGACCTCCAGTTGGCACCAATCGGTAAT |  |
|  |  |  |
| P27 | CTCGTCCGAGGGCAAAGGAATAGAGTAGAAAACAGGCGAAGTATGGTTC | Primers to amplify *BcRIM15* downstream fragment for construction of the gene deletion vector |
| P28 | TTGAGCGCTTGCTGATTCT |  |
|  |  |  |
| P29 | TGTGTCAGGACAATTTGACGG | PCR primers for identification of *BcRIM15* deletion transformants |
| P30 | ATGCGGGGTTTGGAAATTCT |  |
|  |  |  |
| P31 | CGGATTTGTATCCCAGACCTT | Primers to amplify *BcPRO40* upstream fragment for construction of the gene deletion vector |
| P32 | CAAAATAGGCATTGATGTGTTGACCTCCTTTGGTGGATCGATAGGAGTT |  |
|  |  |  |
| P33 | CTCGTCCGAGGGCAAAGGAATAGAGTAGTGAGAAGCAACAGCGTACCAA | Primers to amplify *BcPRO40* downstream fragment for construction of the gene deletion vector |
| P34 | AACCAATACCGCCAAGCTTT |  |
|  |  |  |
| P35 | TCGGAGGGCAACAGACACTAT | PCR primers for identification of *BcPRO40* deletion transformants |
| P36 | AAAGGAGAAATGTTGGCAGG |  |
|  |  |  |
| P37 | AACTACTTCGCCCTCATCATT | Primers to amplify *BcSCH9* upstream fragment for construction of the gene deletion vector |
| P38 | CAAAATAGGCATTGATGTGTTGACCTCCAAACGGATAGAAGAGCGGGAA |  |
|  |  |  |
| P39 | CTCGTCCGAGGGCAAAGGAATAGAGTAGTGAAGCTTATCGGAAAGGGT | Primers to amplify *BcSCH9* downstream fragment for construction of the gene deletion vector |
| P40 | TTGATCCATTCGATCCTCGT |  |
|  |  |  |
| P41 | TCGATTTCGCAATTCTGCA | PCR primers for identification of *BcSCH9* deletion transformants |
| P42 | CTTTGTGAGGTTCGCTTTTG |  |
|  |  |  |
| P43 | AACTAAAAGGCCACCACCAT | Primers to amplify *BcSTE20* upstream fragment for construction of the gene deletion vector |
| P44 | CAAAATAGGCATTGATGTGTTGACCTCCAGTGCGCGCCATCTTCAT |  |
|  |  |  |
| P45 | CTCGTCCGAGGGCAAAGGAATAGAGTAGTGTCTTCCGGGATTTCCTTT | Primers to amplify *BcSTE20* downstream fragment for construction of the gene deletion vector |
| P46 | AGAAGCTAAAGCTCATCCCCA |  |
|  |  |  |
| P47 | CGAAAAAAGAGCAAGAGCAAC | PCR primers for identification of *BcSTE20* deletion transformants |
| P48 | TTTTCTTGTGCTCGGGCATT |  |
|  |  |  |
| P49 | TCTGATGTCAGCTTTTGTGGC | Primers to amplify *BcSTE50* upstream fragment for construction of the gene deletion vector |
| P50 | CAAAATAGGCATTGATGTGTTGACCTCCCGCTGCTACAAATGAAGTCAG |  |
|  |  |  |
| P51 | CTCGTCCGAGGGCAAAGGAATAGAGTAGCAATCACTGTCACCTGGACAA | Primers to amplify *BcSTE50* downstream fragment for construction of the gene deletion vector |
| P52 | TGCTTCAAAGCTGACTGTCT |  |
|  |  |  |
| P53 | CAAGATTAAGCAAGATGTGCC | PCR primers for identification of *BcSTE50* deletion transformants |
| P54 | TTGTTGGTCTGATCGGTACGA |  |
|  |  |  |
| P55 | ACCGACGCAGATACAACCATT | Primers to amplify *BcSTE7* upstream fragment for construction of the gene deletion vector |
| P56 | CAAAATAGGCATTGATGTGTTGACCTCCAGGTGGGGGTGCATTCAA |  |
|  |  |  |
| P57 | CTCGTCCGAGGGCAAAGGAATAGAGTAGTTGTTTATGAACCTGCGCCA | Primers to amplify *BcSTE7* downstream fragment for construction of the gene deletion vector |
| P58 | TGATTTGGTGACGTATCTGCT |  |
|  |  |  |
| P59 | TCACTCATCATCGTTGCCTT | PCR primers for identification of *BcSTE7* deletion transformants |
| P60 | ACGAAAGGTTCACGCTCCTAT |  |
|  |  |  |
| P61 | TGGTTCTGCCGGTCTTTAT | Primers to amplify *BcPACC* upstream fragment for construction of the gene deletion vector |
| P62 | CAAAATAGGCATTGATGTGTTGACCTCC CACCAGCACTTCTTTGGAGTA |  |
|  |  |  |
| P63 | CTCGTCCGAGGGCAAAGGAATAGAGTAGTGTCAGTGACCGACTTCAAAA | Primers to amplify *BcPACC* downstream fragment for construction of the gene deletion vector |
| P64 | TATTTCCAACCCCTCCACAT |  |
|  |  |  |
| P65 | TCTCCAATGTCCTCAAGTGGA | PCR primers for identification of *BcPACC* deletion transformants |
| P66 | CATCATCTGTTTTCAGAACGG |  |
|  |  |  |
| P67 | CGACACCAAAACCCATTCGTA | Primers to amplify *BcATF1* upstream fragment for construction of the gene deletion vector |
| P68 | CAAAATAGGCATTGATGTGTTGACCTCCTGGATCGAAGGCAACAAGGTA |  |
|  |  |  |
| P69 | CTCGTCCGAGGGCAAAGGAATAGAGTAGTGAGTTTCAACGAACGGCTA | Primers to amplify *BcATF1* downstream fragment for construction of the gene deletion vector |
| P70 | GCGAAAAGCATGGTTTTGAG |  |
|  |  |  |
| P71 | ATTACCTTTCCAAGCGAGCGT | PCR primers for identification of *BcATF1* deletion transformants |
| P72 | CATTGTCAGGGTAGTTGCTCA |  |
|  |  |  |
| P73 | AACCCCCCTGGTCTATCTCT | Primers to amplify *BcRLM1* upstream fragment for construction of the gene deletion vector |
| P74 | CAAAATAGGCATTGATGTGTTGACCTCCATCTTTCTTCTCCCCATTGC |  |
|  |  |  |
| P75 | CTCGTCCGAGGGCAAAGGAATAGAGTAG TTGAACCAATGATGCCTCCT | Primers to amplify *BcRLM1* downstream fragment for construction of the gene deletion vector |
| P76 | CGAAGGTGGCTTTATACGACT |  |
|  |  |  |
| P77 | CTTATTTGTGCCGCTCGATT | PCR primers for identification of *BcRLM1* deletion transformants |
| P78 | TGATGGATTTGGCGCATACA |  |
|  |  |  |
| P79 | GATTGAATAGCCCGACTACC | Primers to amplify *BcGIS1* upstream fragment for construction of the gene deletion vector |
| P80 | CAAAATAGGCATTGATGTGTTGACCTCCGGTTCCATTGGGTTGTTCTA |  |
|  |  |  |
| P81 | CTCGTCCGAGGGCAAAGGAATAGAGTAGGATTTGAGCAAGGAGAACCA | Primers to amplify *BcGIS1* downstream fragment for construction of the gene deletion vector |
| P82 | GACACCCCCAGTATAAGAAG |  |
|  |  |  |
| P83 | GGCAGAGAGATGTGAAGAAT | PCR primers for identification of *BcGIS1* deletion transformants |
| P84 | ATCAAGGAAGTGGCCTTTAG |  |
|  |  |  |
| P85 | TACCGACTAACGGTTTCCTT | Primers to amplify *BcMSN2* upstream fragment for construction of the gene deletion vector |
| P86 | CAAAATAGGCATTGATGTGTTGACCTCCCTGGTGTCATAGGTGGAGAC |  |
|  |  |  |
| P87 | CTCGTCCGAGGGCAAAGGAATAGAGTAGCATTACAGATCCCTCCACAC | Primers to amplify *BcMSN2* downstream fragment for construction of the gene deletion vector |
| P88 | TCACATCAATTCGGACACTC |  |
|  |  |  |
| P89 | ATTCTACCCACCCCAGTTA | PCR primers for identification of *BcMSN2* deletion transformants |
| P90 | ATCTTCCAATACGCCCATGA |  |
|  |  |  |
| P91 | GGAGGTCAACACATCAATGCCTATT | PCR primers for amplification of hygromycin resistance gene (*HPH*) |
| P92 | CTACTCTATTCCTTTGCCCT |  |
|  |  |  |
| P93 | GGCATTTCTTTGTATGGCAGG | Primers to amplify *BcOAH* upstream fragment for construction of *BcOAH* and *BcMKK1* double genes deletion mutants |
| P94 | CCAAAATAGCATTGATGTGTTGACCTCCCAAGCGGGAAGCAGTGGTA |  |
|  |  |  |
| P95 | CTATCGCCTTCTTGACGAGTTCTTCTGAATGGCCGCAAAGACTGTT | Primers to amplify *BcOAH* downstream fragment for construction of *BcOAH* and *BcMKK1* double genes deletion mutants |
| P96 | TTCATCAGGTGCCACTGCATA |  |
|  |  |  |
| P97 | AAGTCTAAGGGCACCATTCAA | Primers to amplify *BcRIM15* upstream fragment for construction of *BcRIM15* and *BcMKK1* double genes deletion mutants |
| P98 | CCAAAATAGCATTGATGTGTTGACCTCCAGTTGGCACCAATCGGTAAT |  |
|  |  |  |
| P99 | CTATCGCCTTCTTGACGAGTTCTTCTGAAAAACAGGCGAAGTATGGTTC | Primers to amplify *BcRIM15* downstream fragment for construction of *BcRIM15* and *BcMKK1* double genes deletion mutants |
| P100 | TTGAGCGCTTGCTGATTCT |  |
|  |  |  |
| P101 | AACTACTTCGCCCTCATCATT | Primers to amplify *BcSCH9* upstream fragment for construction of *BcSCH9* and *BcMKK1* double genes deletion mutants |
| P102 | CCAAAATAGCATTGATGTGTTGACCTCCAAACGGATAGAAGAGCGGGAA |  |
|  |  |  |
| P103 | CTATCGCCTTCTTGACGAGTTCTTCTGATGAAGCTTATCGGAAAGGGT | Primers to amplify *BcSCH9* downstream fragment for construction of *BcSCH9* and *BcMKK1* double genes deletion mutants |
| P104 | TTGATCCATTCGATCCTCGT |  |
|  |  |  |
| P105 | GGAGGTCAACACATCAATGCT | PCR primers for amplification of neomycin resistance gene (*NEO*) |
| P106 | TCAGAAGAACTCGTCAAGAAG |  |
|  |  |  |
| P107 | CCATCACATCACAATCGATCCAACCATGCGTCCGGTAATAATTCGAA | PCR primers to amplify *BcBCK1* fragment used for construction of the BcBck1-GFP vector |
| P108 | TACTTACCTCACCCTTGGAAACCATATATGTGCCGCGAATCTTAGCATA |  |
|  |  |  |
| P109 | TAGCCCTGGAACTTTAGGT | PCR primers for identification of ΔBcBck1::BcBck1-GFP (ΔBcBck1-C) transformants |
| P110 | GAGCCATCCTCAATGTTGTG |  |
|  |  |  |
| P111 | CCATCACATCACAATCGATCCAACCATGTCTTCTCCAGCTCCATT | PCR primers to amplify *BcMKK1* fragment used for construction of the BcMkk1-GFP vector |
| P112 | TACTTACCTCACCCTTGGAAACCATCCAGCCCCATACGGTAGC |  |
|  |  |  |
| P113 | AACCCGATGCTGGCATCA | PCR primers for identification of ΔBcMkk1::BcMkk1-GFP (ΔBcMkk1-C) transformants |
| P114 | GAGCCATCCTCAATGTTGTG |  |
|  |  |  |
| P115 | CCATCACATCACAATCGATCCAACCATGGCAGACCTGCAAGGA | PCR primers to amplify *BcBMP3* fragment used for construction of the BcBmp3-GFP vector |
| P116 | TACTTACCTCACCCTTGGAAACCATCGATCGCATAGCATCCAAACCA |  |
|  |  |  |
| P117 | TTCGATTTTGAAGTTGTGGA | PCR primers for identification of ΔBcBmp3::BcBmp3-GFP (ΔBcBmp3-C) transformants |
| P118 | GAGCCATCCTCAATGTTGTG |  |
|  |  |  |
| P119 | CCATCACATCACAATCGATCCAACCATGTTCGAGATCATCAAACC | PCR primers to amplify the protein kinase domain of BcRim15 (BcRim15^PK^) used for construction of the BcRim15^PK^-GFP vector |
| P120 | TACTTACCTCACCCTTGGAAACCATAAACCAAGGATGGCTTCG |  |
|  |  |  |
| P121 | ACTGGTGGTCTGTAGGATGT | PCR primers for identification of BcRim15^PK^-GFP transformants |
| P122 | GAGCCATCCTCAATGTTGTG |  |
|  |  |  |
| P123 | CACCGCGACGTCTGTCGAGAAG | Primers to amplify *HPH* fragment used as the probe for Southern blot analysis |
| P124 | GGACGATTGCGTCGCATCGA |  |
|  |  |  |
| P125 | GGAGGTCAACACATCAATGCT | Primers to amplify *NEO* fragment used as the probe for Southern blot analysis |
| P126 | TCAGAAGAACTCGTCAAGAAG |  |
|  |  |  |
| P127 | TCACGCATTGACACCTACACA | PCR primers for amplification of the *BcCMR1* gene in quantitative real-time PCR assays |
| P128 | TTCCATGTTCCAATTCCTCG |  |
|  |  |  |
| P129 | TCAACCACACGGACCATATC | PCR primers for amplification of the *BcPKS13* gene in quantitative real-time PCR assays |
| P130 | CGCCATCACCAAGAAGACC |  |
|  |  |  |
| P131 | TGCCCGCTTCCCAATTCAT | PCR primers for amplification of the *BcSCD1* gene in quantitative real-time PCR assays |
| P132 | TCCATCACCCTCATTCCATCT |  |
|  |  |  |
| P133 | GATTGAATTCACATATCAAAC | PCR primers for amplification of the *BcCHS1* gene in quantitative real-time PCR assays |
| P134 | TGTAAACATGTGCCATGGGTCG |  |
|  |  |  |
| P135 | TGGGCAACAATCTCTTCACT | PCR primers for amplification of the *BcCHS2* gene in quantitative real-time PCR assays |
| P136 | TTCAGACGGCATTTCGAGTT |  |
|  |  |  |
| P137 | GCTTTCAGTAATTGGCACGA | PCR primers for amplification of the *BcCHS3a* gene in quantitative real-time PCR assays |
| P138 | CCAACAACGCGTTTGAGAAT |  |
|  |  |  |
| P139 | GCACAAAGGGCTCAGATGAAA | PCR primers for amplification of the *BcCHS3b* gene in quantitative real-time PCR assays |
| P140 | TTACCTCTTCGCAAAGCAGTG |  |
|  |  |  |
| P141 | CTTGCTACTTTTGGGAGAGGA | PCR primers for amplification of the *BcCHS4* gene in quantitative real-time PCR assays |
| P142 | TTGTGAAAGCAATAGCGGCT |  |
|  |  |  |
| P143 | ACCCAACAAGATGATGATGGA | PCR primers for amplification of the *BcCHS5* gene in quantitative real-time PCR assays |
| P144 | GACCAAAATATTGACTTCCGC |  |
|  |  |  |
| P145 | CCGAGCAATCCAAATCGAA | PCR primers for amplification of the *BcCHS6* gene in quantitative real-time PCR assays |
| P146 | TCAAGATTGGTTTCGCCACA |  |
|  |  |  |
| P147 | TTGATCATTAATGGTCCCGC | PCR primers for amplification of the laccase gene BC1G_08553 in quantitative real-time PCR assays |
| P148 | ATTTCTTACCACCACCAACGC |  |
|  |  |  |
| P149 | TGGGTTCCAATGATGAGTCTG | PCR primers for amplification of the protease gene BC1G_06849 in quantitative real-time PCR assays |
| P150 | AAGATCGGCTGGGAGAGCAAT |  |
|  |  |  |
| P151 | GTGGTTCATTCTCCGGAAAA | PCR primers for amplification of the protease gene BC1G_03070 in quantitative real-time PCR assays |
| P152 | TTGACTCTTGAGGTTGTCCAA |  |
|  |  |  |
| P153 | TACTGCTGAGCTCCTCGCTTT | PCR primers for amplification of the protease gene BC1G_05845 in quantitative real-time PCR assays |
| P154 | CCCCACTGTAACATTTGCAT |  |
|  |  |  |
| P155 | TGACATTCAATCCCTACCGCT | PCR primers for amplification of the peroxidase gene BC1G_12545 in quantitative real-time PCR assays |
| P156 | TCTTCACCTCCACATCTCTCC |  |
|  |  |  |
| P157 | GAGCAATCAATTGACAGGCA | PCR primers for amplification of the peroxidase gene BC1G_02806 in quantitative real-time PCR assays |
| P158 | AGCAGCCGGTAGCAATTGTT |  |
|  |  |  |
| P159 | ATGCAACTCGCTGTTTGAGA | PCR primers for amplification of the peroxidase gene BC1G_14974 in quantitative real-time PCR assays |
| P160 | CCAGTGGAAGAGTTTGTGCTA |  |
|  |  |  |
| P161 | TTGAAGGTCGCTCGGGAGAT | PCR primers for amplification of the *BcOAH* gene in quantitative real-time PCR assays |
| P162 | AGGTTCTTTGGTGTTCCAACG |  |
|  |  |  |
| P163 | AACCATCTGTCTTGGGTCTTG | PCR primers for amplification of the *BcACTIN* gene in quantitative real-time PCR assays |
| P164 | TGGTGCAATGATCTTGACCT |  |
|  |  |  |
| P165 | GTACCAGATTACGCTCATATGATGCGTCCGGAAACTTTTAAAG | PCR primers for amplification of full *BcBKC1* for construction of the vector pGADT7-BcBkc1 |
| P166 | ATGCCCACCCGGGTGGAATTCTTAATATGTGCCGCGAATCTTAGC |  |
|  |  |  |
| P167 | TCAGAGGAGGACCTGCATATGATGCGTCCGGAAACTTTTAAAG | PCR primers for amplification of full *BcBKC1* for construction of the vector pGBKT7-BcBkc1 |
| P168 | TCGACGGATCCCCGGGAATTCTTAATATGTGCCGCGAATCTTAGC |  |
|  |  |  |
| P169 | GTACCAGATTACGCTCATATGATGTCTTCTCCAGCTCCATTATTACG | PCR primers for amplification of full *BcMKK1* for construction of the vector pGADT7-BcMkk1 |
| P170 | ATGCCCACCCGGGTGGAATTCTCACCAGCCCCATACGGTAG |  |
|  |  |  |
| P171 | TCAGAGGAGGACCTGCATATGATGTCTTCTCCAGCTCCATTATTACG | PCR primers for amplification of full *BcMKK1* for construction of the vector pGBKT7-BcMkk1 |
| P172 | TCGACGGATCCCCGGGAATTCTCACCAGCCCCATACGGTAG |  |
|  |  |  |
| P173 | GTACCAGATTACGCTCATATGATGGCAGACCTGCAAGGAAG | PCR primers for amplification of full *BcBMP3* for construction of the vector pGADT7-BcBmp3 |
| P174 | ATGCCCACCCGGGTGGAATTCCTACGATCGCATAGCATCCAAA |  |
|  |  |  |
| P175 | TCAGAGGAGGACCTGCATATGATGGCAGACCTGCAAGGAAG | PCR primers for amplification of full *BcBMP3* for construction of the vector pGBKT7-BcBmp3 |
| P176 | TCGACGGATCCCCGGGAATTCCTACGATCGCATAGCATCCAAA |  |
|  |  |  |
| P177 | GCCATGGAGGCCAGTGAATTCATGGAGGGAGAGCAGAATCAGG | PCR primers for amplification of full *BcRIM15* for construction of the vector pGADT7-BcRim15 |
| P178 | ATGCCCACCCGGGTGGAATTCTTACCGATCTCTCTTTAAACTTTTACCC |  |
|  |  |  |
| P179 | TCAGAGGAGGACCTGCATATGATGGCAGGAGCGGAACAAG | PCR primers for amplification of full *BcSCH9* for construction of the vector pGBKT7-BcSch9 |
| P180 | TCGACGGATCCCCGGGAATTCTCAATGGAAGTCATCACCATTGA |  |
|  |  |  |
| P181 | TCAGAGGAGGACCTGCATATGATGGCTCGGCGCACTGGA | PCR primers for amplification of full *BcPRO40* for construction of the vector pGBKT7-BcPro40 |
| P182 | TCGACGGATCCCCGGGAATTCTCATCTGCCAAACTCCAAACTG |  |
|  |  |  |
| P183 | TCAGAGGAGGACCTGCATATGATGGCTCGGCGCACTGGA | PCR primers for amplification of partial *BcPRO40* for construction of the vector pGBKT7-BcPro40 (N domain) |
| P184 | TCGACGGATCCCCGGGAATTCTGGTGGAGATGACGGACCAG |  |
|  |  |  |
| P185 | TCAGAGGAGGACCTGCATATGATGGCTCGGCGCACTGGA | PCR primers for amplification of partial *BcPRO40* for construction of the vector pGBKT7-BcPro40 (N+WW domain) |
| P186 | TCGACGGATCCCCGGGAATTCGCCCTTGGGAAATTCCCA |  |
|  |  |  |
| P187 | TCAGAGGAGGACCTGCATATGATGGCTCGGCGCACTGGA | PCR primers for amplification of partial *BcPRO40* for construction of the vector pGBKT7-BcPro40 (N+C domain) |
| P188 | TTAGTGGAGTAGGTGGTGGAGATGACGGACCAG |  |
|  |  |  |
| P189 | GCCATGGAGGCCAGTGAATTCCCGTCATCTCCACCACCGC | PCR primers for amplification of partial *BcPRO40* for construction of the vector pGADT7-BcBmp3 (WW+C domain) |
| P190 | CAGCTCGAGCTCGATGGATCCTCATCTGCCAAACTCCAAACTG |  |
|  |  |  |
| P191 | TCCCCGAATTCCCGGGTCGACATGTCTTCTCCAGCTCCATTATTACG | PCR primers for amplification of full BcMkk1 cDNA for construction of the vector pGEX-4T-3-BcMkk1 |
| P192 | GATGCGGCCGCTCGAGTCGACTCACCAGCCCCATACGGTAG |  |
|  |  |  |
| P193 | TCCCCGAATTCCCGGGTCGACATGGCAGGAGCGGAACAAG | PCR primers for amplification of full BcSch9 cDNA for construction of the vector pGEX-4T-3-BcSch9 |
| P194 | GATGCGGCCGCTCGAGTCGACTCAATGGAAGTCATCACCATTGA |  |
|  |  |  |
| P195 | TAAGAAGGAGATATACATATGATGTTCGAGATCATCAAACCTATCAG | PCR primers for amplification of the protein kinase domain of BcRim15 (BcRim15^PK^) for construction of the vector pET-22b(+)-BcRim15^PK^ |
| P196 | GGTGGTGGTGGTGGTGCTCGAGAAACCAAGGATGGCTTCG |  |
|  |  |  |
| P197 | TGGTTCCGCGTGGATCCCCG | PCR primers for identification of the plasmids of GST-tagged protein |
| P198 | CAGTCAGTCACGATGCGGCC |  |
|  |  |  |
| P199 | GGAATTGTGAGCGGATAAC | PCR Primers for identification of the plasmids of His6-tagged protein |
| P200 | CCTTTCGGGCTTTGTTAGC |  |
