# Supplementary material for: The MAPK kinase BcMkk1 suppresses oxalic acid biosynthesis via impeding phosphorylation of BcRim15 by BcSch9 in Botrytis cinerea
Source: PLoS Pathog. 2018 Sep 13;14(9):e1007285. doi: 10.1371/journal.ppat.1007285 (PMC6136818; doi:10.1371/journal.ppat.1007285)
Supplement: S2 Table — (DOCX) [file ppat.1007285.s009.docx]

**Table S2** A list of partial BcMkk1-interacting proteins identified by affinity capture in coupling with mass spectrometry.

| Proteins | Putative functions | Homologs in  *Saccharomyces cerevisiae* |
| --- | --- | --- |
| Bcin03g07190.1 | MAPKK involved in the protein kinase C signaling pathway | Mkk1/2 |
| Bcin15g03580 | Mitogen-activated protein kinase involved in osmostic regulation | Hog1 |
| Bcin04g02750 | GTP-binding protein regulates protein kinase C | Rho1 |
| Bcin09g02430.1 | Serine/threonine MAP kinase involved in CWI pathway | Slt2 |
| Bcin02g06590.1 | Serine/threonine MAP kinase kinase kinase involved in CWI pathway | Bkc1 |
| Bcin01g06080 | Presumable scaffold protein for the CWI pathway in filamentous fungi | / |
| Bcin12g04680 | Serine/threonine kinase, essential for cell wall remodeling during growth | Pkc1 |
| Bcin15g00280 | Protein kinase involved in cell proliferation in response to nutrients | Rim15 |
| Bcin08g03660 | Adaptor protein for various signaling pathways | Ste50 |
| Bcin13g03980 | Cdc42p-activated signal transducing kinase | Ste20 |
| Bcin11g06110 | Kinase, phosphorylated by Tor1p, required for ribosome biogenesis | Sch9 |
